# Identification of target genes regulated by encystation-induced transcription factor Myb2 using knockout mutagenesis in *Giardia lamblia*

**DOI:** 10.1186/s13071-022-05489-z

**Published:** 2022-10-07

**Authors:** Juri Kim, Eun-Ah Park, Mee Young Shin, Soon-Jung Park

**Affiliations:** Department of Environmental Medical Biology and Institute of Tropical Medicine, Yonsei University College of Medicine, Seoul, 03722 Korea

**Keywords:** *Giardia lamblia*, CRISPR/Cas9-mediated knockout, *G. lamblia* Myb2, Encystation

## Abstract

**Background:**

Encystation is one of the two processes comprising the life cycle of *Giardia lamblia*, a protozoan pathogen with tetraploid genome. *Giardia lamblia* Myb2 (GlMyb2) is a distinct encystation-induced transcription factor whose binding sites are found in the promoter regions of many encystation-induced genes, including its own.

**Methods:**

Two sequential CRISPR/Cas9 experiments were performed to remove four *glmyb2* alleles. The expression level of *G. lamblia* cyst wall protein 1 (GlCWP1), a well-known target gene of GlMyb2, was measured via western blotting and immunofluorescence assays. Chromatin immunoprecipitation experiments using anti-GlMyb2 antibodies were performed on the encysting *G. lamblia* cells. Quantitative real-time PCR was performed to confirm an expression of candidate GlMyb2-regulated genes by comparing the transcript level for each target candidate in wild-type and knockout mutant *Giardia*. The promoter region of *glcwp1* was analyzed via deletion and point mutagenesis of the putative GlMyb2 binding sites in luciferase reporters.

**Results:**

Characterization of the null *glmyb2* mutant indicated loss of functions related to encystation, i.e. cyst formation, and expression of GlCWP1. The addition of the wild-type *glmyb2* gene to the null mutant restored the defects in encystation. Chromatin immunoprecipitation experiments revealed dozens of target genes. Nineteen genes were confirmed as GlMyb2 regulons, which include the *glmyb2* gene, six for cyst wall proteins, five for signal transduction, two for transporter, two for metabolic enzymes, and three with unknown functions. Detailed analysis on the promoter region of *glcwp1* defined three GlMyb2 binding sites important in its encystation-induced expression.

**Conclusions:**

Our data confirm that GlMyb2 acts as a transcription activator especially during encystation by comparing the *glmyb2* knockout mutant with the wild type. Further investigation using *glmyb2* null mutant will provide knowledge regarding transcriptional apparatus required for the encystation process of *G. lamblia*.

**Graphical Abstract:**

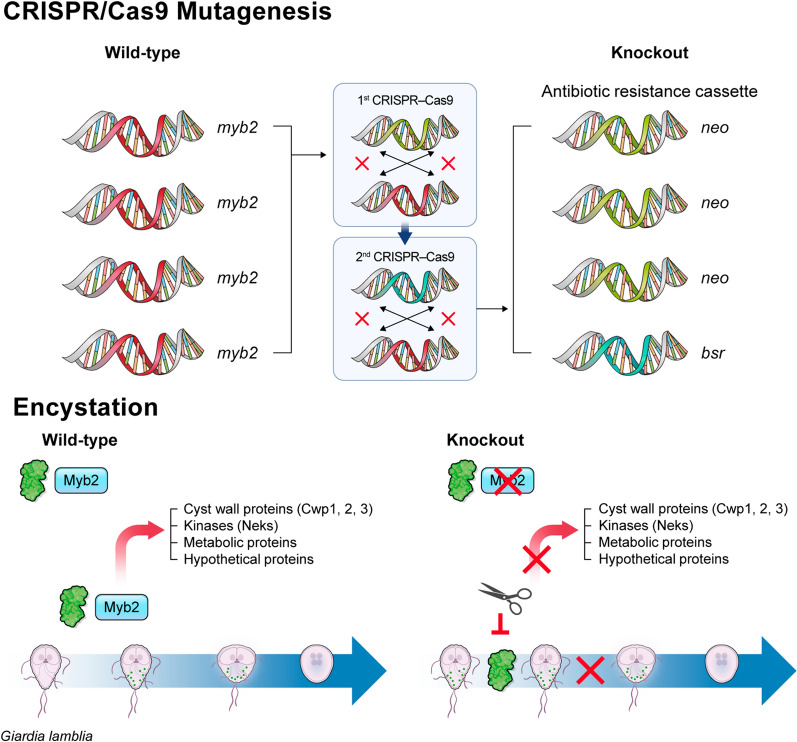

**Supplementary Information:**

The online version contains supplementary material available at 10.1186/s13071-022-05489-z.

## Background

*Giardia lamblia*, a pathogen that causes giardiasis in humans [1], is an interesting protozoan because it has simplified and degenerated organelles, such as the mitosome, and has two or four nuclei with multiple genomes [2]. It has a simple life cycle, comprising trophozoites (vegetative stage) and cysts (dormant stage). Trophozoites inhabit the human small intestine, where they adhere, divide, and cause diseases. Before being released from the human body, they differentiate into cysts, non-dividing but infective forms, via the encystation process triggered by different physiological elements, including alkaline pH, bile concentration, and lipid components [3], and unidentified factors.

Encystation is one of the two key differentiation processes in the *Giardia* life cycle (see [4] for a review). It has been investigated more extensively than other processes, such as excystation, owing to its higher efficiency for in vitro reconstitution. Encystation-induced genes have been investigated at transcriptomic or proteomic levels from the cells in the encystation process induced in vitro or occurring in vivo. RNA samples were prepared from *Giardia* cells treated with two different in vitro encystation protocols and were subsequently used for a comparative microarray analysis. The result showed only 18 overlapping genes with upregulated expression, including genes for cyst wall proteins, glycan synthesis, and a transcription factor, *G. lamblia* Myb2 (GlMyb2) [5]. *Giardia* proteomes at various time points (early, middle, and late stages of encystation) have been examined and documented [6]. The encystation process was reinforced to make cysts more efficient, and the related transcriptomes at various time-points were investigated using RNA-seq [7]. An animal infection model using bioluminescence-tagged *G. lamblia* demonstrated that this pathogen colonizes the gut in high-density foci where the expressions of encystation-related proteins are upregulated [8]. Close examination of the encystation process using deep RNA-seq led to the categorization of encystation proteins according to the time points showing their maximal expressions [9].

One of the distinct encystation-induced proteins was GlMyb2, as previously reported [10, 11], and putative GlMyb2 binding sites were found in the promoter regions of most of the encystation-induced genes [5]. The binding of GlMyb2 to the promoters of genes involved in cyst formation was detected via in vitro gel shift assays and in vivo chromatin immunoprecipitation (ChIP) experiments [12]. In addition, several transcription factors have been identified as regulators of the expression of *G. lamblia* cyst wall proteins (GlCWPs), such as ARID/bright-like protein [13], WRKY protein [14], Pax proteins [15, 16], and E2F [17].

It has been difficult to develop genetic tools for knockout mutagenesis because *Giardia* trophozoites have tetraploid genomes [18, 19]. Using Causes recombination-locus of crossing over in P1 system, four copies of the GlCWP1 (*glcwp1*) locus were removed, resulting in a pseudocyst upon encystation [20]. The clustered, regularly interspaced short palindromic repeat (CRISPR)/Cas9 system, originally discovered as an adaptive immune system of prokaryotes (see [21] for a review), has been repurposed for genome editing in a broad range of model organisms from yeast to mammalian cells [22]. In *G. lamblia*, the gene manipulation techniques for gene knockout using CRISPR/Cas9 have been developed to investigate the role of myeloid leukemia factor (MLF) protein in *glcwp* expression and cyst formation [23]. Knockout mutant *G. lamblia* in type 1A DNA topoisomerase (TOP3β) was made via the application of CRISPR/Cas9 and demonstrated decreased expressions of *glcwp1-3* and *glmyb2* genes [24]. In addition, CRISPR/Cas9 system-mediated knockdown experiments resulted in mutant *Giardia* with a partial loss of the gene encoding a multi-protein bridging factor 1-like protein (MBF1) [25]. The CRISPR system using nuclease-dead Cas9, instead of Cas9, was developed and successfully knocked down the expression of motor proteins, kinesin-2a and kinesin-13, and a ventral disc protein [26]. Recently, Horáčková et al. [27] attempted genome editing using an in vitro assembled CRISPR/Cas9 component, but they had no success. Instead, they made a knockout mutant of three genes [*mem* (multiple high cysteine membrane), *cwp1* and *mlf1*] using a Cas9-expressing cell line.

The first goal of this study was to remove the encysting-specific transcription factor gene *glmyb2* using CRISPR/Cas9 based on homologous recombination. We also aimed to identify the GlMyb2 target genes using ChIP-seq and verify them by comparing the expressions of the target promoters in the wild-type and *glmyb2* knockout mutant. These studies will provide clues to understand the underlying mechanisms of the encystation process.

## Methods

### Cultivation and induction of in vitro encystation of Giardia lamblia trophozoites

*Giardia lamblia* trophozoites (strain WB, ATCC30957; American Type Culture Collection, Manassas, VA, USA) were grown in modified TYI-S-33 medium (2% casein digest, 1% yeast extract, 1% glucose, 0.2% NaCl, 0.2% l-cysteine, 0.02% ascorbic acid, 0.2% K_2_HPO_4_, 0.06% KH_2_PO_4_, pH 7.1) supplemented with 10% heat-inactivated calf serum (Sigma-Aldrich, St. Louis, MO, USA) and 0.5 mg/ml bovine bile at 37 °C [28] and used as wild type. Transfected and mutant *Giardia* strains were cultured in TYI-S-33 medium containing appropriate antibiotics at the following concentrations (50 µg/ml puromycin, 600 µg/ml G418, and 75 µg/ml blasticidin).

In vitro encystation was induced as previously described [29]. Briefly, *Giardia* cells at an exponential phase were incubated in a TYI-S-33 medium with 10 mg/ml bovine bile at pH 7.8. At various time points (0, 3, 6, 14, and 24 h), *Giardia* cells were harvested for quantitative PCR (qPCR), and immunofluorescence assays (IFAs).

### Construction of CRISPR/Cas9 systems

Two CRISPR/Cas9 systems were constructed to generate *glmyb2* knockout mutant *Giardia* based on a study by McInally et al. [26] in which *Streptococcus pyogenes* Cas9 (SpCas9) was expressed in a chimeric form with both nuclear localization signal sequences (NLSs) of SV40 and those of a hypothetical protein of *G. lamblia* (GL50803_2340). The DNA fragment encoding SpCas9 with SV40 NLSs was amplified from pRGEN-Cas9-CMV (ToolGen, Seoul, Korea) by PCR using primers, SpCas9-F and SpCas9-R, and then cloned into the *Not*I and *Xba*I sites of pPgdh-3HA.PAC [30] to produce pSpCas9.PAC. Another DNA fragment encoding the NLS of the open reading frame (ORF) of GL50803_2340 was amplified from *Giardia* genomic DNA using primers 2340-F and 2340-R and then cloned into the *Xba*I and *Eco*RI sites of pSpCas9.PAC to generate pSpCas9NLS.PAC.

Four DNAs encoding guide RNAs (gRNAs) were synthesized and used to delete the *glmyb2* gene in *G. lamblia* genome. Three of the four gRNA sequences (RG1, RG2, RG3) were designed by a company (ToolGen), and the remaining gRNA (RG4) was made using the CRISPR RGEN Tools (http://www.rgenome.net/cas-designer/). Based on the information provided by McInally et al. [26], a spliceosomal snRNA expression cassette, pUC57-gRNA, was synthesized as a gRNA expression plasmid in *G. lamblia* (GenScript, Piscataway, NJ, USA). Oligonucleotides encoding four gRNAs and a control RNA were cloned into the *Bbs*I site of pUC57-gRNA, resulting in gRNA expression plasmids pUC-Cont, pUC-RG1, pUC-RG2, pUC-RG3, and pUC-RG4.

The neomycin (*neo*) resistance cassette was amplified from pKS-3HA.NEO [31] using primers neo-cassette-F and neo-cassette-R. The upstream and downstream regions of the *glmyb2* gene (1 kb each) were amplified by PCR using primers myb2-up-F/myb2-up-R and myb2-down-F/myb2-down-R, respectively. The resulting DNA fragments were cloned into the *Apa*I and *Not*I sites of pBluescript II SK to generate pSK-mybNEO. The DNA fragment containing the upstream and downstream regions of the *glmyb2* gene with the *neo* cassette in the center was amplified by PCR using the primers myb2NEO-F and myb2NEO-R. This was subsequently cloned into the *Xba*I and *Hind*III sites of each gRNA expression plasmid using infusion ligation system (TAKARA, Shiga, Japan) to produce the following plasmids: pCont-mybNEO, pRG1-mybNEO, pRG2-mybNEO, pRG3-mybNEO, and pRG4-mybNEO.

The up- and downstream regions (1 kb each) of the *glmyb2* gene were amplified by PCR using primers 2nd-myb2-up-F/2nd-myb2-up-R and 2nd-myb2-down-F/2nd-myb2-down-R, respectively, to construct the second CRISPR/Cas9 system. The resulting DNA and *neo* cassettes were cloned into pUC-RG1 to generate pRG1-dmybNEO. The linearized pRG1-dmybNEO plasmid lacking the *neo* cassette was amplified by PCR using primers dmyb-F and dmyb-R. The DNA fragment encoding the blasticidin-S resistance (*bsr*) cassette was amplified from pKS-3HA.BSR [31] by PCR using primers bsr-F and bsr-R. The resulting DNA fragments were ligated to produce pRG1-dmybBSR.

All plasmids and primers used are described in Additional file 1: Table S1 and Additional file 2: Table S2, respectively.

### Generation of glmyb2 knockout mutant strain

Plasmid pSpCas9NLS.PAC was transfected into *Giardia* trophozoites by electroporation under the following conditions: 700 Ω, 1000 μF, and 300 V. SpCas9 expression was confirmed by western blotting, and nuclear localization of SpCas9 in the transfectants was detected by IFAs. The linearized form of one of the following plasmids containing gRNA and *neo*-resistance cassette with the upstream and downstream regions of the *glmyb2* gene (pCont-mybNEO, pRG1-mybNEO, pRG2-mybNEO, pRG3-mybNEO, and pRG4-mybNEO) was transfected into *Giardia* trophozoites carrying pSpCas9NLS.PAC. The transfectants were seeded in 96-well microtiter plates (5 cells/well) with G418 (600 μg/ml) selection. After three limiting dilutions of the G418-resistant cells in microtiter plates, the *glmyb2* locus of these cells was analyzed by PCR using the primers myb2-Det-F and myb2-Det-R. The cells demonstrating both wild-type and mutant *glmyb2* loci were induced to undergo encystation, and their GlCWP1 expression was determined by western blot analysis.

The second CRISPR/Cas9 system (pSpCas9NLS.PAC and pRG1-dmybBSR) was transfected into *Giardia* cells via electroporation as mentioned above. The transfectant cells were selected for blasticidin (75 μg/ml) resistance via three sequential limited dilutions, as described above. Blasticidin-resistant cells were examined by PCR using primers Det2-F and Det2-R to confirm the deletion of the remaining *glmyb2* gene. The candidate cells were analyzed by whole-genome sequencing. The genotypes of all strains are described in Additional file 1: Table S1.

### Western blot analysis

Vegetative or encysting cells were harvested and resuspended in lysis buffer (50 mM NaH_2_PO_4_, 300 mM NaCl, and 10 mM imidazole, pH 8.0) containing a 1% protease inhibitor cocktail (GenDEPOT, Katy, TX, USA). Extracts prepared from various *Giardia* cells (i.e. cells carrying the SpCas9 expressing plasmid, *glmyb2* knockout strains, and cells carrying the complementation plasmid) were separated by sodium dodecyl sulfate-polyacrylamide gel electrophoresis and transferred onto a polyvinylidene fluoride membrane (Millipore, Bedford, MA, USA). The membrane was incubated with mouse monoclonal anti-SpCas9 (1:500; Abcam, Cambridge, MA, USA), mouse monoclonal anti-HA (1:1,000; Sigma-Aldrich), or rat polyclonal anti-GlCWP1 (GL50803_5638, 1:10,000; [32]) antibodies in Tris-buffered saline with Tween 20 solution (50 mM Tris–HCl, 5% skim milk and 0.05% Tween 20) at 4 °C overnight. The membranes were subsequently incubated with horseradish peroxidase-conjugated secondary antibodies and immunoreactive proteins were visualized using an enhanced chemiluminescence system (Thermo Fisher Scientific, Waltham, MA, USA). Next, the membranes were incubated in the stripping buffer (Thermo Fisher Scientific) at room temperature for 20 min and reacted with polyclonal rat antibodies against protein disulfide isomerase 1 (PDI1; GL50803_29487) of *G. lamblia* (1:10,000) as the loading control [33].

### Immunofluorescence assay

*Giardia* cells were attached to glass slides coated with l-lysine for 10 min, followed by treating with ice-cold methanol for 10 min and permeabilizing with phosphate-buffered saline (PBS; 137 mM NaCl, 2.7 mM KCl, 10.1 mM Na_2_HPO_4_ and 2 mM KH_2_PO_4_, pH 7.4)/0.5% Triton X-100 for 10 min. After blocking for 1 h in PBS/5% goat serum/3% bovine serum albumin (BSA), the cells were incubated with the primary antibodies in PBS/3% BSA overnight at 4 °C and treated with fluorescent dye-conjugated secondary antibodies afterwards. The samples were mounted with ProLong™ Gold Antifade Mountant with DAPI (Thermo Fisher Scientific) and observed using an inverted confocal laser scanning microscope (LSM700; Carl Zeiss, Oberkochen, Germany). The following antibodies were used at the indicated dilutions: anti-SpCas9 mouse monoclonal antibodies (1:100; Abcam), rat anti-GlCWP1 polyclonal antibodies (1:200; [32]), Alexa Fluor 488-conjugated goat anti-mouse IgG (1:100; Thermo Fisher Scientific), and Alexa Fluor 555-conjugated goat anti-rat IgG (1:200; Thermo Fisher Scientific).

### Whole-genome sequencing

Genomic DNAs (gDNAs) were prepared from the *glmyb2* gene knockout strains using MG Genomic Extraction Kit (MGmed, Seoul, Korea). The integrity of gDNA was checked by agarose gel electrophoresis, and the amount of gDNA was quantified using Quant-IT PicoGreen (Invitrogen, Carlsbad, CA, USA). Sequencing libraries were prepared according to the manufacturer’s instructions using the TruSeq DNA Nano Library Prep Kit (Illumina, San Diego, CA, USA). Briefly, 100 ng of gDNA was fragmented using adaptive focused acoustic technology (Covaris, Woburn, MA, USA), and the fragmented DNA was then end-repaired to produce the 5′-phosphorylated and blunt-ended double-stranded DNA molecules. They were fractionated based on their size of 150 bp by adding a single ‘A’ base to these DNA fragments prior to being ligated with the Truseq indexing adapters. The libraries were quantified using qPCR according to the qPCR Quantification Protocol Guide (KAPA Library Quantificatoin kits for Illumina Sequencing platforms) and tested for quality using the Agilent Technologies 2200 TapeStation (Agilent Technologies, Santa Clara, CA, USA). Sequencing was performed by the company (Macrogen, Seoul, Korea), using the NovaSeq platform (Illumina).

### Scanning electron microscopy

*Giardia* cells were pre-fixed in a Karnovsky fixative solution (2% glutaraldehyde, 2% paraformaldehyde in phosphate buffer, pH 7.4), washed, and post-fixed with 1% osmium tetroxide. The samples were dehydrated in an ascending gradual series (50–100%) of absolute ethanol and used in a critical point dryer (Leica EM CPD300, Leica Microsystems, Wetzlar, Germany). For observation by a scanning electron microscope, the dehydrated samples were coated with an ion coater (Leica EM ACE600, Leica Microsystems) and observed under a field emission scanning electron microscope (MERLIN, Carl Zeiss).

### Cyst counting

Stationary phase culture cells were seeded at 2 × 10^4^ cells/ml in a 10-ml tube and grown for 48 h until approximately 80% confluence. The medium was discarded, and the attached cells were cultured in the encysting medium for 48 h. After 48 h, the tubes were chilled on ice for 20 min, and the cells were harvested. The harvested cells were washed once with PBS and incubated in either cold PBS or double-distilled water overnight at 4 °C. The numbers of cells in PBS and water-resistant cysts in double-distilled water were counted using a hemocytometer for WB, JK1, and JK2. The percentage of cyst formation was calculated based on the ratio of the number of water-resistant cysts in distilled water to the total number of cells in PBS. Three independent experiments were performed with three separate cultures.

### Construction of the glmyb2 complementation strain

A 1590-bp fragment DNA containing the *glmyb2* gene and 150 bp of the upstream region was amplified by PCR using primers, Pmyb2-F and myb2-R, and cloned into the *Not*I/*Xho*I site of pKS-3HA.PAC to produce pPmyb2HA.PAC. This plasmid was transfected into the *glmyb2* knockout mutant *G. lamblia* by electroporation. As a control, an empty vector, pKS-3HA.PAC was transfected into the WB or the *glmyb2* knockout mutant *Giardia*. The expression of the GlMyb2 protein in these cells was examined by western blotting using anti-HA antibodies. The level of *glmyb2* transcript was determined by qPCR. All primers and plasmids used are described in Additional file 2: Table S2 and Additional file 1: Table S1, respectively.

### Quantitative PCR

Total RNA was prepared from *Giardia* WB carrying an empty vector, JK2, carrying an empty vector, and JK2 carrying the *glmyb2* complementation plasmid using TRIzol (Thermo Fisher Scientific) according to the manufacturer’s instructions. Five micrograms of RNA were converted into complementary DNA using the Improm-II Reverse Transcription System (Promega, Madison, WI, USA). The qPCR was performed using the LightCycler 480 SYBR Green I Master Kit (Roche Applied Science, Basel, Switzerland). The conditions for qPCR were as follows: pre-incubation for 5 min at 95 °C and 45 cycles of 30 s at 94 °C, 30 s at 56 °C, and 30 s at 72 °C. The nucleotide sequences of the forward and reverse primers used for real-time PCR are listed in Additional file 2: Table S2. The *G. lamblia* actin-related gene (*glactin*; GL50803_15113) transcript was used to normalize the amount of mRNA in the samples, which was constitutively expressed during the cell cycle of *G. lamblia* [34]. Relative quantifications of these data were determined by measuring the crossing-point value using the Light Cycler 480 II real-time PCR system software program (Roche Applied Science, version LSC480 1.5.0.39). The results were expressed as means ± standard deviations of three independent experiments. All experiments were performed with three separate cultures.

### Antibody generation

A 1590-bp *glmyb2* DNA fragment was amplified by PCR using the primers rmyb2-F and rmyb2-R and then cloned into the *Sac*I/*Not*I site of pET32a to produce pET-myb2. Histidine-tagged GlMyb2 was overexpressed in *Escherichia coli* BL21 (DE3) by adding 1 mM IPTG at 37 °C. The resulting recombinant proteins were excised from the SDS-PAGE and used to immunize Sprague-Dawley rats (2 weeks old, female; Orient Bio, Seongnam, Korea) to produce polyclonal antibodies, as previously described [35]. The rats received humane care according to our institutional guidelines and the legal requirements (institutional approval number: 2012–0264-1).

### Chromatin immunoprecipitation sequencing

*Giardia lamblia* WB cells were grown in a TYI-S-33 medium to approximately 70% confluence and cultured in the encysting medium. The cells harvested at 3, 6, and 14 h post-encystation were treated with 0.75% formaldehyde at room temperature for 10 min. The reactions were stopped by adding 125 mM glycine, and the harvested cells were washed with ice-cold PBS. The fixed cells were resuspended in ChIP buffer (140 mM NaCl, 1 mM EDTA, 1% Triton X-100, 0.1% sodium deoxycholate, 1.0% SDS, 50 mM HEPES–KOH, pH 7.5 and protease inhibitor cocktail) and then stored at –80 °C until use.

Cell extracts prepared by sonication were incubated with rat anti-GlMyb2 antibodies in RIPA buffer (50 mM Tris–HCl, 150 mM NaCl, 2 mM EDTA, 1% NP-40, 0.5% sodium deoxycholate, 0.1% SDS, pH 8.0, and protease inhibitor cocktail) for 2 h at 4 °C. Next, the protein A/G beads pre-treated with salmon sperm DNA/BSA were added into the reaction. The beads were sequentially washed with a low-salt wash buffer (0.1% SDS, 1% Triton X-100, 2 mM EDTA, 20 mM Tris–HCl, 150 mM NaCl, pH 8.0), a high-salt wash buffer (500 mM NaCl), and an LiCl wash buffer (250 mM LiCl, 1% NP-40, 1% sodium deoxycholate, 1 mM EDTA, 10 mM Tris–HCl, pH 8.0). Eluted samples with the elution buffer (1% SDS, 100 mM NaHCO_3_) at 30 °C for 15 min were subsequently treated with 200 mM NaCl and 160 μg/ml RNase A overnight at 65 °C. The samples were then incubated with 420 μg/ml proteinase K for 1 h at 60 °C. Chromatin extracts without anti-GlMyb2 antibodies were also prepared as controls.

After purification using the PCR Purification Kit (Nucleogen, Seoul, Korea), the eluted DNA was processed for ChIP-sequencing (Macrogen). Briefly, the quantity and quality of the DNA samples were evaluated by using Quant-IT PicoGreen (Invitrogen) and the Agilent 2100 Bioanalyzer (Agilent Technologies), respectively. The sequencing libraries were prepared according to the manufacturer’s instructions using the TruSeq ChIP Sample Preparation Kit (Illumina) and then sequenced using the HiseqX™ platform (Illumina) by Macrogen.

### Construction of luciferase reporters and determination of luciferase activity

A 516-bp DNA fragment encoding nanoluciferase was amplified by PCR using the primers Nluc-F and Nluc-R from the plasmid pNL1.1 (Promega). Linearized pKS-3HA.PAC was prepared by PCR using primers KS-F and KS-R. These two DNA fragments were ligated via infusion cloning to produce pNluc.PAC. Promoter DNAs of the following genes (150–200 bp) were amplified from *Giardia* gDNA by PCR using the primers listed in Additional file 2: Table S2. Then, they were cloned into either *Sac*I/*Hind*III sites or *Not*I/*Pst*I sites in pNluc.PAC: *glcwp1* (GL50803_5638), *glcwp2* (GL50803_5435), and *glcwp3* (GL50803_2421), a gene encoding high cysteine non-variant cysteine protein (GL50803_40376), and a gene encoding acetyl-CoA carboxylase/pyruvate carboxylase (GL50803_113021).

The *glcwp1* promoter regions, 25 bp, 50 bp, 75 bp, and 100 bp upstream of the GlCWP1 ORF, were amplified by PCR using pP5638-Nluc.PAC as a template, and cloned into pNluc.PAC to produce pP5638-25, pP5638-50, pP5638-75, and pP5638-100 plasmids. These constructs were transfected into the WB by electroporation, and the transfected cells were selected using 50 μg/ml puromycin.

The 75-bp promoter region of *glcwp1* was amplified by PCR with 5638-mt1-F and 5638-R primers using pP5638-75 as a template and then cloned into pNluc.PAC. The resulting plasmid, pP5638-mt1, contained the luciferase gene under the control of the *glcwp1* promoter with a mutation at the GlMyb2 binding site [5, 10]. The same procedure was conducted for pP5638-mt2 and pP5638-mt3 using 5638-mt2-F/5638-R and 5638-mt3-F/5638-R, respectively. Other plasmids, pP5638-mt4 and pP5638-mt5, were constructed by PCR using pP5638-100 as the template with 5638-mt4-F/5638-R and 5638-mt5-F/5638-R, respectively. These constructs were transfected into *Giardia* WB by electroporation, and the transfected cells were selected using 50 μg/ml puromycin. All the primer sequences are listed in Additional file 2: Table S2. All constructs were verified by DNA sequencing (Macrogen).

Luciferase activity in *Giardia* WB cells carrying these fusions was determined using the Luciferase Assay System (Promega) with a luminometer (TD-20/20 Luminometer, Turners Designs, San Jose, CA, USA). Specific bioluminescence was calculated by normalizing the relative light units to the protein concentration.

### Statistical analyses

Data are presented as means ± standard error of data from three independent experiments. Statistical analysis was performed using Student’s *t*-test (Systat Program, SigmaPlot version 9; Systat Software, Inc.) to determine the statistical significance of these results. Differences with *P* values are indicated in the figures by one asterisk (*P = 0.01–0.05) or two asterisks (**P < 0.01).

## Results

### The glmyb2 gene was deleted by two series of CRISPR/Cas9-mediated mutagenesis in G. lamblia

The *glmyb2* (GL50803_8722) gene, which encodes a transcription factor showing encystation-induced expression [10, 11], was chosen as the target gene for genome editing using the CRISPR/Cas9 system. We first established *Giardia* expressing SpCas9 in the nucleus. Based on the information provided by a previous study [26], a plasmid pSpCas9NLS.PAC was constructed in which SpCas9 was expressed from *G. lamblia* glutamate dehydrogenase promoter (*glgdh*, GL50803_21942) as a chimeric form with an NLS of another protein of *Giardia* (GL50803_2340) and SV40 NLS at its C-terminus (Additional file 3: Fig. S1a). Upon transfection with this plasmid, SpCas9 expression was detected by western blot analysis using anti-SpCas9 antibodies as an immunoreactive band of 160 kDa (Additional file 3: Fig. S1b). *Giardia* carrying an empty vector did not show this band. The amount of protein in the extracts was confirmed by monitoring the level of *G. lamblia* protein disulfide isomerase 1, GlPDI1 (GL50803_29487). Finally, the expression of SpCas9 in *Giardia* nuclei was demonstrated by IFA using anti-SpCas9 antibodies (Additional file 3: Fig. S1c).

**Fig. 1:**
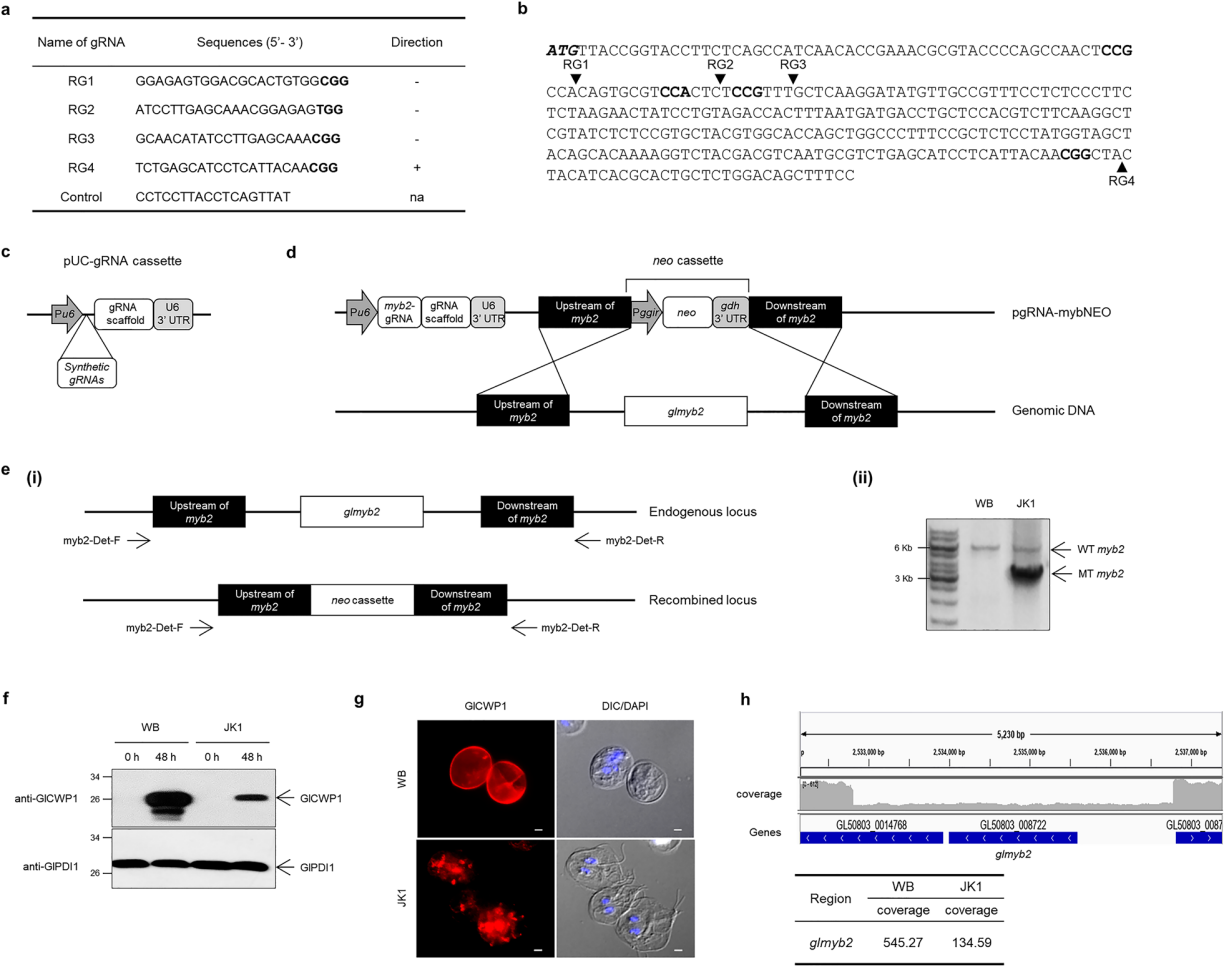
The first CRISPR/Cas9-mediated mutagenesis of *G. lamblia myb2* (*glmyb2*) gene. **a** Nucleotide sequences encoding the guide RNAs (gRNAs) for *glmyb2* mutagenesis. Control gRNA is made of random nucleotide sequences, which do not trigger SpCas9 nuclease activity. The nucleotides in bold letters indicate the protospacer adjacent motif (PAM) sequence recognized by SpCas9. **b** Target sites cleaved by SpCas9 within the *glmyb2* coding region (GL50803_8722). The cleaved site by each gRNA is indicated with an arrowhead with the SpCas9 recognition sites in bold. The initiation codon of GlMyb2 is represented in bold and italic letters. **c** A schematic diagram of gRNA expression cassette for *G. lamblia*. **d** A schematic diagram for the first CRISPR/Cas9 mutagenesis of the *glmyb2* gene. This plasmid encodes gRNA for SpCas9-mediated cleavage in the *glmyb2* gene and the adjacent regions of the *glmyb2* gene and neomycin resistance cassette, which triggers the deletion of the *glmyb2* gene and replacement with a neomycin resistance cassette. **e** Generation of the *glmyb2* mutant via the first CRISPR/Cas9 mutagenesis. (**i**) Schematic figures for wild-type and mutant *glmyb2* genes in *G. lamblia* genome. Primers used to probe the *glmyb2* locus in the candidate cells are indicated. (**ii**) PCR analysis of the *glmyb2* locus of wild-type (WB) and mutant JK1. **f** Western blot analysis of WB and mutant JK1 using anti-GlCWP1 antibodies. Both WB and mutant JK1 incubated in the encystation medium for 48 h were used to prepare cell extracts to determine the level of GlCWP1. **g** An immunofluorescence assay of WB and JK1 strains as trophozoites and 24 h-encysting cells using anti-GlCWP1 antibodies. Scale bars, 2 μm. **h** Coverage plot of the target gene region on *Giardia* chromosome 5 at positions 51,829 through 55,605. The blue bars indicate gene annotations from GeneDB. Read alignment views of the *glmyb2* gene region of whole genome sequence and an average depth of fragment reads

We designed four guide RNAs (gRNAs; RG1, RG2, RG3, and RG4) (Fig. 1a), which were predicted to cleave the indicated sites within the *glmyb2* gene (Fig. 1b). Random DNA oligonucleotides [26] were used as a control in this study (Fig. 1a). DNA oligonucleotides for each gRNA including control oligonucleotides were cloned into a gRNA expression cassette (Fig. 1c). The resulting gRNA cassette was used to produce the pgRNA-mybNEO plasmid, which contained the *neo* cassette between the up- and downstream regions of the *glmyb2* gene (Fig. 1d). Trophozoites carrying the pCont-mybNEO plasmid encoding control gRNA were converted into cysts with approximately 50% lower efficiency than that of WB (data not shown).

After the resulting plasmids were transfected into *Giardia* cells carrying pSpCas9NLS.PAC, the transfectants were selected for neomycin resistance via three sequential limiting dilutions. Genomic DNAs prepared from the resulting cells were examined for the status of their *glmyb2* locus using PCR. PCR with myb2-Del-F and myb2-Del-R could predict a single DNA of either 6 or 3.2 kb, representing the wild-type and mutant *glmyb2* loci, respectively [Fig. 1e (i)]. However, PCR with cells carrying pgRNA-mybNEOs showed either a single DNA of 6 kb or two DNA bands at 6 and 3.2 kb (data not shown for RG1, RG2, and RG3; for RG4, see Additional file 4: Fig. S2a). WB carrying pCont-mybNEO demonstrated only a PCR product of 6 kb, indicating the wild-type *glmyb2* locus (Additional file 4: Fig. S2b). None of the cells demonstrated a mutant PCR product without the wild type. Among *Giardia* cells showing two PCR products, 2–4 lines were further examined for the expression of GlCWP1 during encystation (Fig. 1f, g). The cells with lower level of GlCWP1 were selected and named JK1 [Fig. 1e (ii)]. The JK1 genome was analyzed using whole-genome sequencing. While the other regions were the same as the WB, the incidence of the *glmyb2* locus in JK1 was one-fourth of that of the WB. This result indicated that three out of four *glmyb2* copies were missing in JK1 (Fig. 1h).

**Fig. 2:**
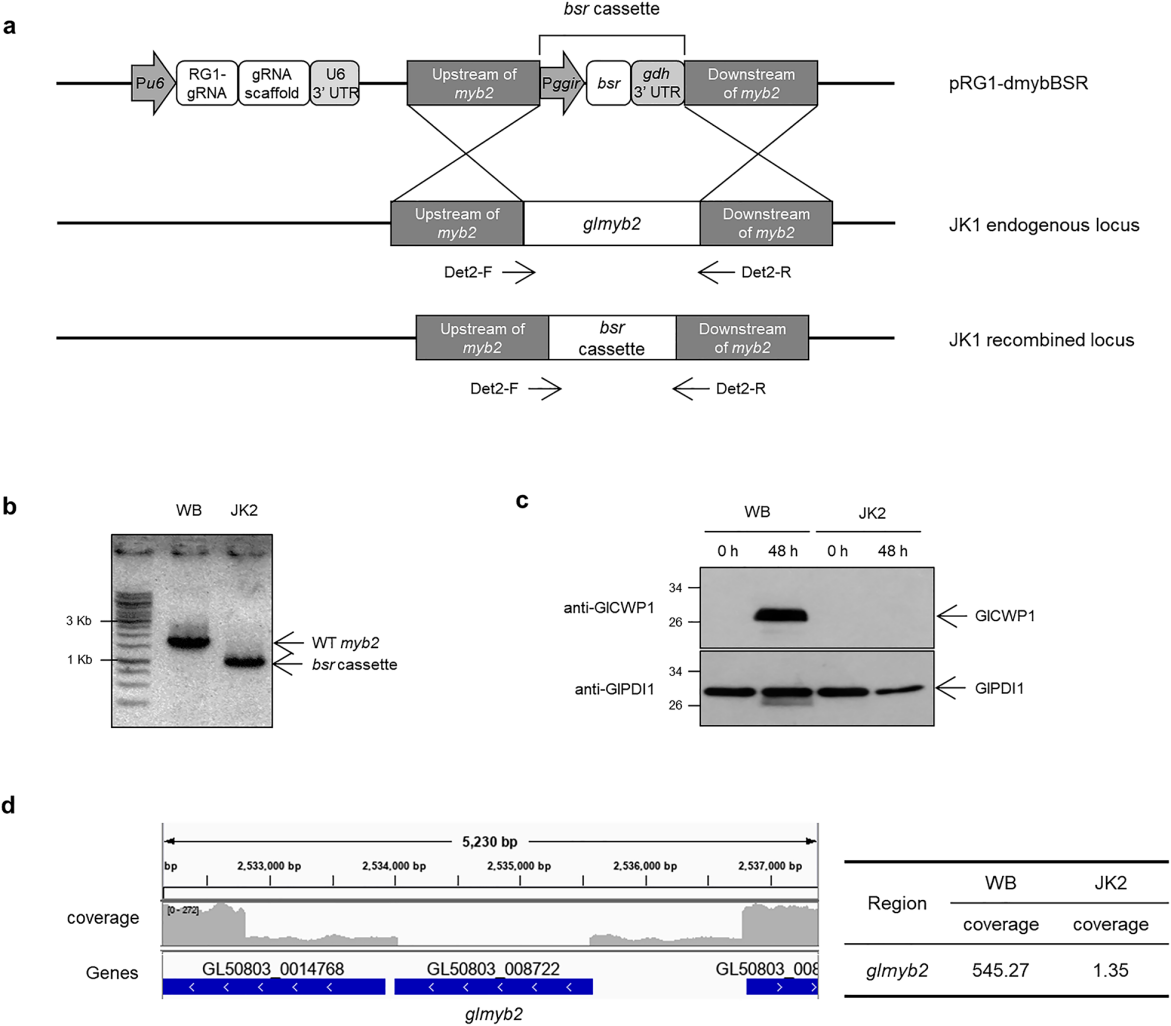
The second CRISPR/Cas9-mediated mutagenesis of the wild-type *glmyb2* gene in JK1. **a** Schematics showing the strategy to delete the remaining wild-type *glmyb2* gene in JK1. The plasmid used for mutagenesis of the JK1 strain contains the blasticidin-S resistance (*bsr*) cassette between the up- and downstream regions of the *glmyb2* gene and RG1 gRNA expression cassette. This plasmid was transfected into JK1 by electroporation, and the transfectants were selected with three series of limiting dilutions with 75 μg/ml of blasticidin. **b** PCR analysis of the *glmyb2* locus of wild-type (WB) and mutant JK2. **c** Western blot analysis of WB and mutant JK2 *Giardia* using anti-GlCWP1 antibodies (1:10,000). Both WB and mutant JK2 incubated in the encystation medium for 48 h were used to prepare cell extracts to determine the level of GlCWP1. The same membrane was treated in stripping buffer and then reacted with antibodies specific to GlPDI1 (1:10,000). **d** Coverage plot of the target gene region on *Giardia* chromosome 5 at positions 51,829 through 55,605. The blue bars indicate gene annotations from GeneDB. Read alignment views of the *glmyb2* gene region of whole genome sequence and an average depth of fragment reads

An additional CRISPR/Cas9 system with blasticidin resistance and RG1 gRNA expression cassettes was constructed and used to mutagenize JK1 (Fig. 2a) to remove the remaining *glmyb2* copy in JK1. In this system, the up- and downstream regions of *glmyb2* in this plasmid were located closer to the GlMyb2 ORF than those in pRG1-mybNEO. Therefore, this system may undergo homologous recombination only with the wild-type *glmyb2* locus present in JK1. Screening the transfectants by PCR using Det2-F and Det2-R resulted in *glmyb2* knockout mutant cells showing a PCR product of 831 bp named JK2 (Fig. 2b). The expression of GlCWP1 was dramatically affected in the encysting JK2, as shown by western blotting with anti-GlCWP1 antibodies (Fig. 2c). The JK2 genome was analyzed using whole-genome sequencing (Fig. 2d). The other regions besides the adjacent sequences of *glmyb2* were the same as those of the wild type. The outermost portions of the adjacent sequences of *glmyb2* were one fourth of those of the wild-type DNA as in JK1 (Fig. 1h). The middle portion of the DNA, including the GlMyb2 ORF, was barely detected in JK2, demonstrating that all four *glmyb2* alleles were deleted in JK2.

### The glmyb2 deletion mutants showed a dramatic defect in glcwp1 expression and cyst formation during encystation

**Fig. 3:**
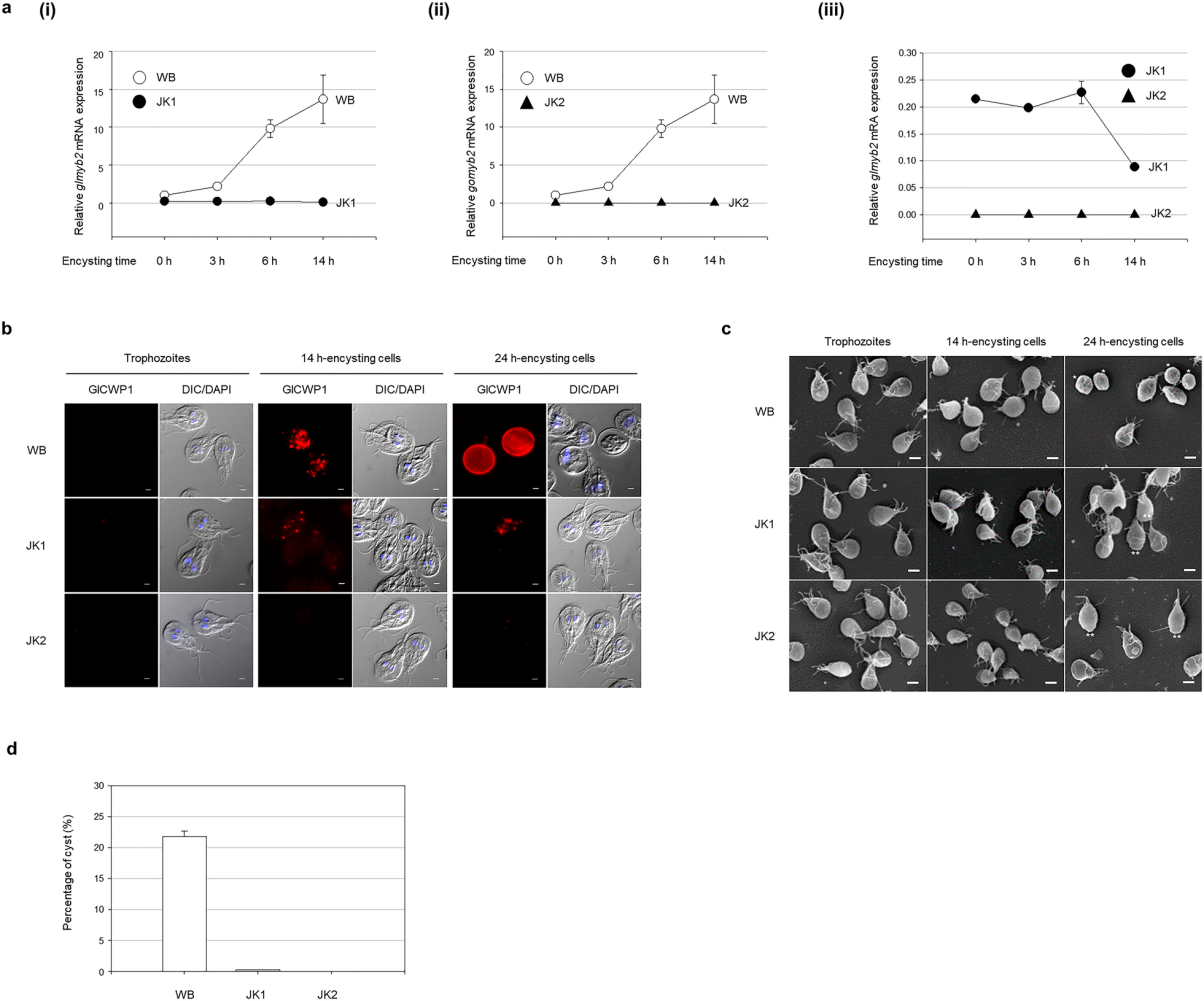
The *glmyb2*-deletion mutants JK1 and JK2 show defective *glcwp1* expression and cyst formation during encystation. **a** Quantitative PCR (qPCR) analysis of *glmyb2* gene expression during encystation. Various *Giardia* cells (WB, JK1, and JK2) incubated in the encystation medium for 3, 6, and 14 h were used to extract RNA samples. qPCR was performed using primers specific for *glmyb2* genes, and transcript levels were normalized to those of the *G. lamblia* actin-related gene (*glactin*; GL50803_15113). Three independent replicates, each consisting of three technical replicates, were evaluated. Error bars represent the standard error of the mean. **b** IFAs of WB, JK1, and JK2. *Giardia* trophozoite, 14 h and 24 h encysting cells were stained with anti-GlCWP1 antibodies (1:200) and then incubated with Alexa Fluor 555-conugated anti-rat IgG (1:100). The slides were mounted with ProLong™ Gold Antifade Mountant with DAPI. They were then observed with an Axiovert 200 fluorescent microscope. Differential interference contrast (DIC) image shows cell morphology. Scale bars, 2 μm. **c** Cyst formation of WB, JK1, and JK2 strains. Scanning electron micrograms of various *Giardia* strains (WB, JK1, and JK2) as trophozoites and encysting cells at 14 h and 24 h post-induction to encystation. Cysts are indicated with asterisks, and pseudocyst phenotype cells are indicated with double asterisks. The bars indicate 2 μm. **d** Efficiency of wild-type and mutant *Giardia* in cyst formation. WB, JK1, and JK2 strains were incubated in encystation medium for 48 h and treated with water, and then their cyst formation was analyzed. Three independent experiments were performed and analyzed. Error bars represent the standard error of the mean

The WB and two *glmyb2* mutant cells, JK1 and JK2, were examined for their intracellular levels of *glmyb2* transcript in vegetative and encysting stages by qPCR (Fig. 3a). As expected, the level of *glmyb2* transcript in the WB was significantly increased during encystation. Both *glmyb2* transcripts were hardly detectable in the RNA samples of the JK1 and JK2 strains. A closer comparison between JK1 and JK2 demonstrated that some *glmyb2* transcripts were present in JK1, whereas the expression of *glmyb2* was not found in JK2. Expression and localization of GlCWP1 during encystation were also examined in *glmyb2* mutant strains JK1 and JK2 as well as in the WB by IFAs (Fig. 3b). In the WB cells, GlCWP1 was not detected in trophozoites but was found in the encystation-specific vesicles (ESVs) at 14 h post-induction to encystation. At a later phase of encystation, the wild type was found as cysts where GlCWP1 was present in the cell walls. For JK1, GlCWP1 was found in ESVs but less frequently than in the WB cells. Cyst formation was hardly detected in JK1 cells of 24 h post-induction to encystation while GlCWP1 was present in ESVs in these cells. In contrast, ESVs were not observed in any of the encysting JK2 cells. The morphology of these cells was examined using a scanning electron microscopy. Cysts were found only among the wild-type cells at 24 h post-induction to encystation. Contrarily, the mutant cells JK1 and JK2 did not form cysts while they maintained structural features of trophozoites. Some encysting JK1 and JK2 cells showed a pseudocyst phenotype with smooth cell walls (Fig. 3c) as described [20]. The efficiency of cyst formation in these mutant cells was also evaluated as indicated by the number of cysts derived from 48 h encysting cells (Fig. 3d). Twenty-two percent of WBs (8.1 × 10^6^ cells) were changed into cysts while < 1% (0.3%) of JK1 cells (1.1 × 10^7^ cells) were cysts. Nonetheless, cyst formation was hardly detected in JK2 cells (1.1 × 10^7^ cells).

### Complementation of wild-type glmyb2 gene to the glmyb2 deletion mutant rescued the defects in the encystation process of G. lamblia

**Fig. 4:**
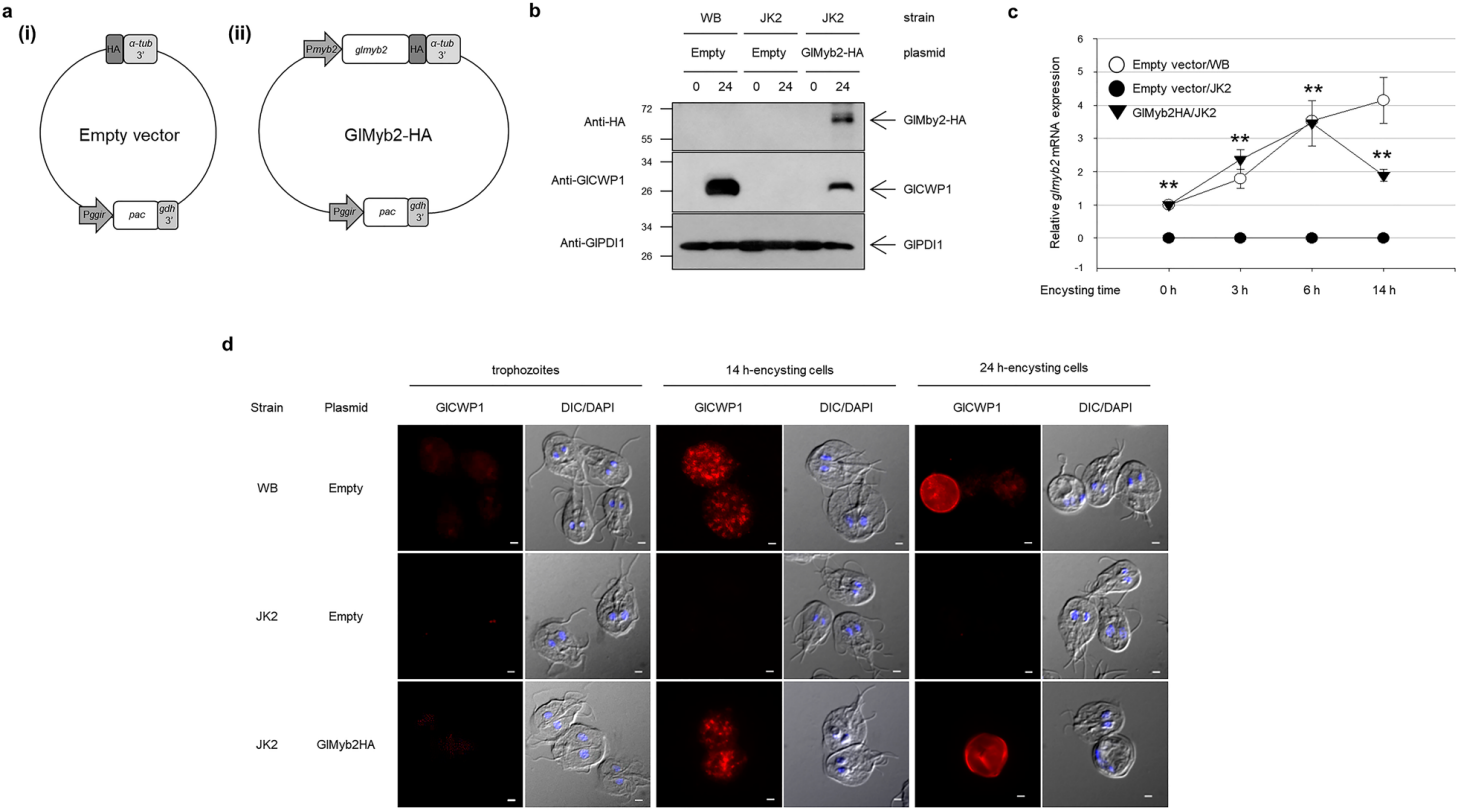
Complementation of the JK2 strain with the wild-type *glmyb2* gene. **a** Diagrams of an empty vector and the complementation plasmid. (**i**) Empty vector contains a *pac* gene (puromycin resistance) under the control of a promoter of the gamma-giardin gene (P*ggir*) and 3′-untranslated region (3′-UTR) of the glutamate dehydrogenase gene (*gdh*). This plasmid has the DNA encoding the hemagglutinin (HA) tag with 3′-UTR of α-tubulin gene (*α-tub*). (**ii**) The complementation plasmid pPmyb2HA.PAC contains the *glmyb2* gene with its own promoter (P_*glmyb2*_), and GlMyb2 is expressed in an HA-tagged C-terminus form. **b** Western analysis. Protein extracts were prepared from trophozoites and 24 h encysting cells of various *Giardia* cells (WB carrying an empty vector, JK2 with an empty vector, and JK2 with the complementation plasmid) and then reacted with anti-HA antibodies. The blot was subsequently incubated with anti-GlPDI1. **c** Quantitative PCR (qPCR) to measure the level of *glmyb2* transcript. Various *Giardia* cells (WB carrying an empty vector, JK2 with an empty vector, and JK2 with the complementation plasmid) incubated in encystation medium for 3, 6, and 14 h, were used to extract RNA samples. qPCR was performed using primers specific for *glmyb2* genes, and transcript levels were normalized to those of the *G. lamblia actin-related gene*, *glactin* (GL50803_15113). Relative *glmyb2* mRNA expression in various cells. Three independent replicates, each consisting of three technical replicates, were evaluated. Error bars represent the standard error of the mean. **P < 0.01. **d** IFAs showing GlCWP1 expression in various *Giardia* cells during encystation. Trophozoite, 14 h, 24 h encysting cells were stained with anti-GlCWP1 antibodies and then incubated with Alexa Fluor 555-conjugated anti-rat IgG. The slides were mounted with ProLong™ Gold Antifade Mountant with DAPI. They were then observed with an Axiovert 200 fluorescent microscope. Differential interference contrast (DIC) image shows cell morphology. Scale bars, 2 μm

A complementation experiment was performed to confirm that the defects of JK2 in encystation were caused by the absence of the *glmyb2* gene (Fig. 4). A complementation plasmid, pGlMyb2-HA, was constructed in which the wild-type GlMyb2 was expressed from its own promoter in an HA-tagged form (Fig. 4a) and subsequently transfected into JK2, the *glmyb2* knockout mutant. As a control, pKS-3HA.PAC, the vector plasmid for pGlMyb2-HA, was transfected into the WB or JK2 cells. The expression of HA-tagged GlMyb2 was detected in the complemented *Giardia*, that is, JK2 with pGlMyb2-HA (Fig. 4b). The level of *glmyb2* transcript was also monitored in these *Giardia* cells (WB with an empty vector, JK2 with an empty vector, and JK2 with the complementation plasmid) by quantitative real-time PCR (Fig. 4c). The complementation strain, JK2 with pGlMyb2-HA, showed a similar level of expression as the control cells (WB with an empty vector) except at the period of 14 h post-induction until encystation. Interestingly, the level of *glmyb2* transcript in the complemented *Giardia* at 14 h post-induction to encystation was lower than that of control cells, WB with the empty vector. The expression of *glmyb2* has been reported to be autoregulated in a positive manner [10, 11], and it is possible that the difference in the levels of GlMyb2 between these two cells caused the differential expression levels of *glmyb2* transcript.

According to the western blotting with anti-GlCWP1 antibodies, we demonstrated that the complementation strain expressed GlCWP1 but at a lower level than the WB carrying an empty vector (Fig. 4b). GlCWP1 was not observed in JK2 cells transfected with an empty vector. Expression of HA-tagged GlMyb2 in JK2 cells partially restored the ability of *Giardia* cells to produce GlCWP1. IFAs of the complementation cells using anti-GlCWP1 antibodies showed formation of ESVs and cyst at 14 h and 24 h encystation, respectively (Fig. 4d). Neither ESVs nor cysts were detected in JK2 cells transfected with an empty vector. Wild-type *Giardia* with the empty vector produced ESVs that gave rise to cyst wall during encystation.

### Isolation of target genes by ChIP-seq and comparative transcriptional analysis in wild-type versus glmyb2 knockout mutant confirmed the genuine GlMyb2 regulons during encystation in G. lamblia

In the following experiment, GlMyb2 regulons were found by the interaction of their promoters with GlMyb2. Compared to ChIP-seq data of *Giardia* trophozoites, those on the encysting cells at 3 h or 6 h post-encystation did not provide any meaningful clones. The cells at 14 h post-encystation demonstrated 51 clones with a > 1.5-fold increase of incidence, which were then categorized according to their putative functions (Table 1). Two of them were found to be the promoter region of *glmyb2* gene showing a five- to sixfold increase compared to those of trophozoites, confirming the autoregulation of GlMyb2 as previously reported [10, 11]. In addition, five genes, encoding reverse transcriptase, 28S ribosomal RNA, 5.8S ribosomal RNA, and two hypothetical proteins, were reported more than once in this ChIP-seq.

**Table 1:** List of genes isolated as GlMyb2 regulon by ChIP-sequencing

Gene ID	Protein	Increase fold	Presence of promoter
Structural protein (6)			
GL50803_5435	Cyst wall protein 2	7.7	Yes
GL50803_5638	Cyst wall protein 1	5.4	Yes
GL50803_2421	Cyst wall protein 3	4.1	Yes
GL50803_113531	High cysteine membrane protein EGF-like	5.3	Yes
GL50803_114626	High cysteine membrane protein EGF-like	4.7	Yes
GL50803_40376	High cysteine non-variant cyst protein	3.7	Yes
Transcription factor (1)			
GL50803_8722	Myb 1-like protein	5.9, 4.8	Yes
Stress response (1)			
GL50803_2556	ATP-dependent Clp protease ATP-binding subunit ClpB	4.5	No
Signaling pathway (7)			
GL50803_137701	Kinase, NEK	6.2	Yes
GL50803_21924	Kinase, NEK	6.1	Yes
GL50803_114495	Kinase, NEK	5.7	Yes
GL50803_42657	Kinase, NEK	4.6	Yes
GL50803_16862	Kinase, NEK	4.2	No
GL50803_13109	RabA	3.1	Yes
GL50803_7512	Protein tyrosine phosphatase-like protein	2.7	Yes
Transport (2)			
GL50803_6664	Zinc transporter domain	6.9	Yes
GL50803_15469	SKD1 protein	3.1	Yes
Metabolism (8)			
GL50803_21118	Long chain fatty acid CoA ligase 5	3.6	No
GL50803_112103	Arginine deiminase	3.6	Yes
GL50803_14993	Pyrophosphate-fructose 6-phosphate 1-phosphotransferase alpha subunit	3.3	Yes
GL50803_14651	Glucosamine 6-phosphate N-acetyltransferase	2.7	Yes
GL50803_113021	Acetyl-CoA carboxylase/pyruvate carboxylase fusion protein, putative	2.6	Yes
GL50803_137673	Reverse transcriptase/endonuclease, putative	2.2, 1.9, 1.7, 1.6	No
GL50803_r0021	28S ribosomal RNA	1.8, 1.6	No
GL50803_r0018	5.8S ribosomal RNA	1.7	No
Unknown function (1)			
GL50803_7875	Parkin co-regulated gene	3.0	Yes
Hypothetical protein (18)			
GL50803_115669	Hypothetical protein	5.9	No
GL50803_7107	Hypothetical protein	5.4, 4.0	No
GL50803_846	Hypothetical protein	5.2	No
GL50803_98653	Hypothetical protein	4.5	No
GL50803_14164	Hypothetical protein	4.3, 3.9	No
GL50803_21048	Hypothetical protein	4.2	Yes
GL50803_27724	Hypothetical protein	4.1	No
GL50803_105798	Hypothetical protein	4.0	No
GL50803_112008	Hypothetical protein	3.8	No
GL50803_39607	Hypothetical protein	3.6	No
GL50803_16078	Hypothetical protein	3.6	Yes
GL50803_105640	Hypothetical protein	3.1	No
GL50803_113285	Hypothetical protein	3.1	No
GL50803_98638	Hypothetical protein	3.0	No
GL50803_2926	Hypothetical protein	3.0	Yes
GL50803_36117	Hypothetical protein	2.9	No
GL50803_16731	Hypothetical protein	1.9	No
GL50803_135294	Hypothetical protein	1.6	No

**Fig. 5:**
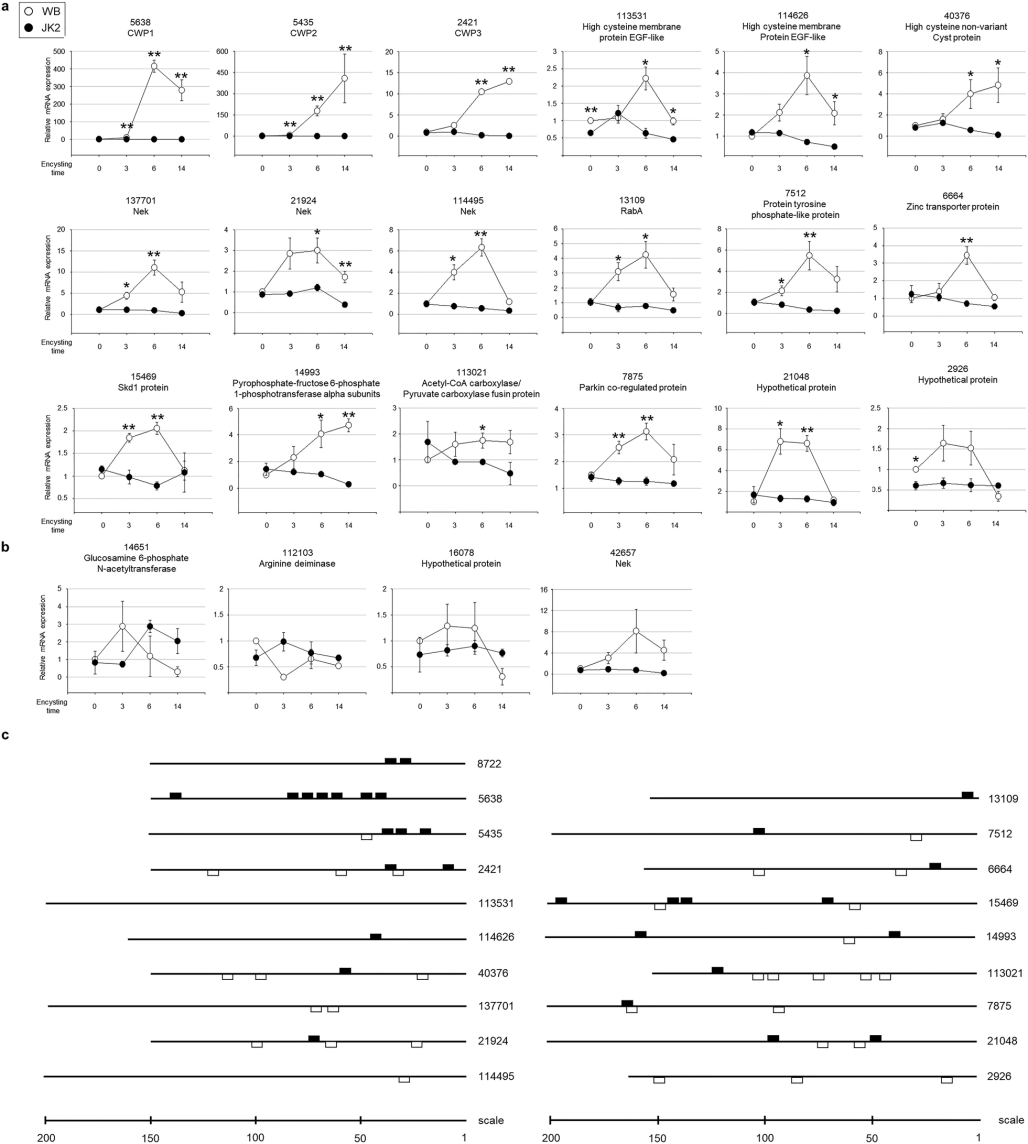
Transcript level determination of candidate GlMyb2 regulons identified by ChIP-seq. Both WB and JK2 strains grown in an encystation medium for 3, 6, and 14 h were harvested for RNA extraction. The transcript levels of 22 candidate genes were monitored in both WB (open circles) and JK2 (closed circles) by quantitative PCR using gene-specific primers. For each gene transcript levels were normalized to those of actin (GL50803_15113). Each gene is indicated by the gene number in a state of omission of GL50803_, provided by the *Giardia* database, and its putative function. **a** GlMyb2 target genes showing GlMyb2-dependent and encystation-induced expression. *P = 0.01–0.05, **P < 0.01. **b** Candidate genes of which expression is independent of encystation and/or GlMyb2. Three independent replicates, each consisting of three technical replicates, were evaluated. Error bars represent the standard error of the mean. **c** Schematic diagrams showing consensus GlMyb2-binding sites in the promoters of the identified GlMyb2 target genes

Since 27 out of the isolated clones did not contain the promoter region, they were excluded from further analysis. Among the 24 clones showing an increased incidence in encysting cells, expressions of two clones with the *glmyb2* promoter region had been examined in both the wild-type/mutant and control cells/complemented cells (Figs. 3a, 4c, respectively). The expression of 18 genes showed an upregulation during encystation and was abolished in the *glmyb2* knockout mutant *Giardia* (Fig. 5a). These included well-known genes that encode components of the cyst wall, namely, *glcwp1*, *glcwp2*, and *glcwp3*. Interestingly, one clone (GL50803_40376) encoding high cysteine non-variant cysteine protein (HCNCP) and two clones encoding high cysteine-rich membrane proteins with epidermal growth factor repeats (HCMPEGF) (GL50803_113531 and GL50803_114626) were found to be target genes of GlMyb2 by showing their increased levels of expression during encystation (Fig. 5a). One of the genes (GL50803_7875) encodes a putative Parkin co-regulated protein with an unknown function. Five clones encoded signaling components: three never-in-mitosis A (NimA)-related kinases (Neks), one Ras-related protein, and one protein tyrosine phosphatase. The expressions of genes encoding transport proteins for zinc and potassium were also regulated by a GlMyb2-mediated pathway. There was no information on the function of two clones annotated with hypothetical proteins. The remaining two clones with GlMyb2-dependent upregulations during encystation were expected to encode metabolic enzymes, including pyrophosphate-fructose 6-phosphate 1-phosphotransferase and acetyl-CoA carboxylase/pyruvate carboxylase. Among the clones examined for their transcriptional levels, the expressions of three genes were not induced during encystation (Fig. 5b). A Nek clone (GL50803_42657) showed an increased expression during encystation in WB, whereas it was decreased in JK2. Nonetheless, the difference was not statistically significant.

Based on the consensus sequences defined by Morf et al. [5], the upstream regions (150–201 bp) of the GlMyb2 target genes were screened for GlMyb2 binding sites (Fig. 5c). This sequence was not found in the promoter regions of one target gene [HCMPEFG (GL50803_113531)]. Only one binding site was proposed in the upstream region of three target genes [HCMPEFG (GL50803_114626), GlNek (GL50803_114495), and RabA (GL50803_13109)]. In contrast, two genes encoding suppressor of K^+^ transport growth defect 1 (SKD1; GL50803_15469) and acetyl-CoA carboxylase/pyruvate carboxylase (GL50803_113021) had six GlMyb2-binding sites. The promoters for *glcwp1-3* had relatively more consensus sequences of GlMyb2, ranging from four to seven. The remaining 10 clones including GlMyb2 (GL50803_8722) contained two to four GlMyb2 binding sites.

### Deletion and mutagenesis in glcwp1 promoter-luciferase reporter defined the role of putative GlMyb2 binding sites

Among the clones showing GlMyb2-mediated induction during encystation in the quantitative real-time PCR data, the promoter regions (150 bp) of five genes were chosen to generate luciferase reporters (Additional file 5: Fig. S3). Both the WB and JK2 cells carrying the empty vector showed low levels of luciferase activity, which did not increase during encystation (Additional file 5: Fig. S3a). Wild-type luciferase reporters for *glcwp1*, *glcwp2*, and *glcwp3* showed an increased activity upon the induction of encystation. The activity of these luciferase reporters was completely abolished when they were transfected into the JK2 cells. For two additional genes encoding HCNCP and acetyl-CoA carboxylase/pyruvate carboxylase fusion protein, the luciferase reporter plasmids were constructed. Their expressions were monitored in trophozoites and during encystation in both WB and JK2 cells (Additional file 5: Fig. S3b). Both genes demonstrated the encystation-induced expressions and the dependency of their activities on GlMyb2.

**Fig. 6:**
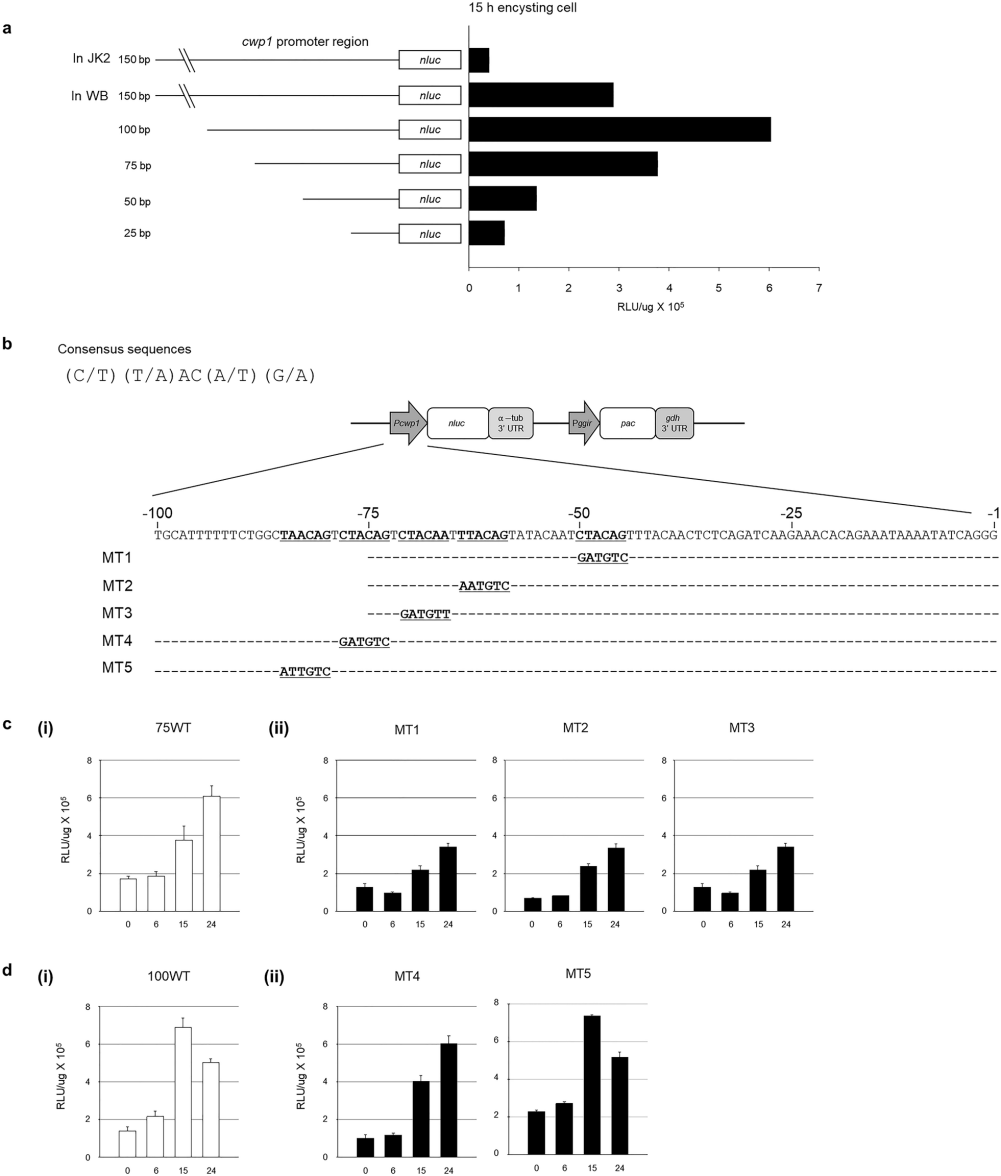
Definition of important cis-acting element(s) in the promoter region of *glcwp1*, P_*glcwp1*_ using luciferase reporter. **a** Expression analysis of a series of luciferase reporters with various sizes of P_*glcwp1*_. P_*glcwp1*_s of various sizes (25–150 bp) were cloned to an empty vector, and the resulting plasmids were transfected to wild-type *Giardia*. Their luciferase activity at 15 h post-induction to encystation was compared. Three independent experiments were performed and analyzed from three experiments. Error bars represent the standard error of the mean. **b** Site-directed mutagenesis of putative GlMyb2-binding sites in the P_*glcwp1*_-luc reporter. Five putative sequences suggested by the GlMyb2 consensus sequence were mutated in the P_*glcwp1*_-luc reporter. Role of putative GlMyb2 binding sites in P_*glcwp1*_ expression (**c**, **d**). **c** Comparison of luciferase activity of the promoter mutants MT1, MT2, and MT3 with that of their wild-type promoter of 75 bp. **d** Comparison of luciferase activity of the promoter mutants MT4 and MT5 with that of their wild-type promoter of 100 bp. Cells carrying these plasmids were incubated in an encystation for 6, 15, and 24 h and used for luciferase assays. Luciferase activity was measured in the presence of luciferin using a luminometer (TD-20210, Turner Designs). Specific bioluminescence was calculated by normalizing the relative light units (RLU) with protein concentration. Three independent replicates, each consisting of three technical replicates, were evaluated. Error bars represent the standard error of the mean

For the *glcwp1* gene, a series of luciferase reporters were constructed, which contained various size of the upstream region of the GlCWP1 ORF, ranging from 25 to 150 bp (Fig. 6a). These constructs were transfected into the WB, and their expression was determined at 15 h post-induction to encystation. The *glcwp1* promoter-luciferase reporter with a shorter upstream region (25–50 bp) showed a low activity level. *Giardia* cells carrying the reporter with 75-bp upstream region of *glcwp1* gene demonstrated an equivalent activity to that of cells with 150-bp promoter. Interestingly, *Giardia* cells with a 100-bp promoter showed a higher level of luciferase activity than those with longer promoter regions of *glcwp1*. This result indicated the importance of cis-acting elements present in the DNA sequence between -50 and -100 relative to + 1 as the start codon of GlCWP1.

Five putative GlMyb2 binding sites in the DNA sequence between -45 and -100 relative to + 1 as the start codon of GlCWP1 [5, 10] were mutated in the *glcwp1*-*luc* reporter plasmid (Fig. 6b). For promoter mutants (MT1, MT2, and MT3), a plasmid containing a 75-bp upstream region was used as a template for mutagenesis. In contrast, the other promoter mutants, MT4 and MT5, were constructed using the plasmid with 100-bp *glcwp1* promoter. None of the mutant promoters resulted in a complete loss of activity (Fig. 6c–d). Even though there was an encystation-induced expression, the fold of induction was significantly decreased in the luciferase reporter with MT1, MT2, and MT3 (Fig. 6c). In contrast, when they were compared with a reporter with 100-bp wild-type upstream region, MT4 and MT5 did not affect the luciferase gene expressions (Fig. 6d). This result indicated that three GlMyb2 binding sites at -45, -59, and -66 relative to + 1, an initiation codon of GlCWP1, had a functional role together in the transcriptional activation of *glcwp1* during encystation by interacting with GlMyb2.

## Discussion

Gene knockout is one of the major strategies used to define gene functions in diverse organisms, including *G. lamblia*. Initially, four copies of the *glcwp1* locus were removed using Causes recombination locus of crossing over in P1 system, resulting in defects during the encystation, which is an intricate and time-consuming process [20]. As a tool for generating knockout mutants of *G. lamblia*, CRISPR/Cas9 has been developed and used to investigate the roles of MLF protein [23], TOP3β [24], and MBF1 [25] in encystation. However, their studies resulted in knockdown but not knockout mutants of *G. lamblia* in which only one or two of the four copies of its tetraploid genome were removed. In mutagenesis of the *mlf* gene, Scr7, an inhibitor for non-homologous end joining pathway, was included to enhance knock-in mutagenesis of drug resistance cassette via the homologous recombination pathway [23]. Since a database search of *G. lamblia* genome indicated that this protozoan does not have components for non-homologous end joining, a homologous recombination-mediated knock-in mutagenesis was performed by introducing the drug marker resistance cassette flanked by upstream and downstream regions of *glmyb2* gene (Figs. 1d, 2a).

The separation of cells with an altered genome from the wild-type cells is the most critical step for mutagenesis. Therefore, the mutant cells obtained using the CRISPR/Cas9 system with *glmyb2neo* cassette were enriched through three sequential limiting dilutions. As the frequency of cells with wild-type/mutant PCR products over wild-type PCR product was the highest in cells transfected with RG1 gRNA, the construct expressing RG1 gRNA was used in the second CRISPR/Cas9 mutagenesis. Because of the unavailability of methodology to differentiate the variable mutants with one, two, or three altered *glmyb2* copies, an additional screening step for detecting the expressions of the *glcwp1* gene resulted in JK1, a knockdown mutant cell, in which three of the four *glmyb2* copies were mutated (Fig. 1f–h). Application of the second CRISPR/Cas9 system to this strain increased a success rate of completely removing the *glmyb2* gene from *Giardia* genome among the transfectants showing blasticidin resistance (Fig. 2a). Although the *glmyb2* knockout mutant was obtained in this study, the knockout mutagenesis of *Giardia* is still difficult owing to the following limitations. Most importantly, the target gene should not be essential for the growth of *Giardia* trophozoites. The *glmyb2* gene was chosen for mutagenesis based on our unproven hypothesis that it may be less important for the vegetative growth of *Giardia* because its expression is induced during encystation. These conditions were not optimized for complete knockout mutagenesis using a single CRISPR/Cas9. In case of mutagenesis using two sequential CRISPR/Cas9 systems, mutant cells obtained from the first CRISPR/Cas9 mutagenesis should have mutation in three of the four alleles. Therefore, methods should be available to differentiate mutant with three deleted alleles from the mutants with one or two altered copies. This study used two CRISPR/Cas9 systems with different drug marker resistance cassettes (neomycin and blasticidin) to generate *glmyb2* knockout strain. The development of drug markers other than neomycin, puromycin, and blasticidin will accelerate research on the improvement of genetic technology in *Giardia*. The knockout methodology recently reported by Horáčková et al. [27] was fundamentally similar as we did. However, they suggested that their method was a highly efficient process for removing non-essential genes (*mem*, *cwp1*, and *mlf1*) in a one-step process and used fluorescence in situ hybridization to identify a deletion mutant. Contrarily, we obtained the *glmyb2* knockout mutant in two-step processes and validated the mutant using a whole-genome sequencing.

As mentioned earlier, *Giardia* encystation has been extensively studied, and the genes with upregulated expression during this process have been well documented [5–9]. Of 18 genes with upregulated expressions during encystation, the promoter regions of 16 genes have putative GlMyb2 binding sites [5]. The expression of many genes with GlMyb2 binding sites in the upstream region was highly upregulated at 7–14 h post-induction to encystation, suggesting that GlMyb2 is the master regulator of encystation [9]. In this study, we unearthed GlMyb2 target genes by ChIP-seq assay using anti-GlMyb2 antibodies, and their authenticity was confirmed using the *glmyb2* knockout mutant. Clones were found more frequently at 14 h post-induction to encystation than in vegetative growth. These were closely examined to determine whether they contained the promoters and monitored their upregulated expressions during encystation (Fig. 5a). Transcript levels of the isolated clones were compared between the wild-type and *glmyb2* knockout mutant cells. Most importantly, the promoter of *glmyb2* was detected by ChIP-seq, indicating that its encystation-induced expression was autoregulated.

Promoters of three abundant cyst wall protein genes, *glcwp1*, *glcwp2*, and *glcwp3*, were found more frequently detected in ChIP-seq of encysting cells, and their expression was upregulated during encystation in a GlMyb2-dependent manner. The interaction between GlMyb2 and promoters of these *glcwp* genes has been demonstrated via ChIP assays and knockdown experiments for *glmyb2* genes [36]. The HCNCP clone (GL50803_40376) found in this study was reported to be upregulated during encystation [37]. The expression of six HCMPEGFs (GL50803_95162, GL50803_113038, GL50803_114815, GL50803_16477, GL50803_16322, and GL50803_8687) was upregulated during encystation [38]. In our study, two additional HCMPEGFs (GL50803_114626 and GL50803_3531) were target genes of GlMyb2, emphasizing that their expressions were induced by encystation (Fig. 5a; Table 1).

In addition to the structural components of cyst stated above, several metabolic enzymes were found as GlMyb2-regulons. The GlMyb2-target gene (GL50803_113021) encodes a large enzyme, a fused form of acetyl-CoA carboxylase/pyruvate carboxylase fusion protein in *G. lamblia*. The expression of this protein was previously found to be upregulated at 24 h of encystation [9]. In our ChIP-seq and transcript analyses, an additional metabolic enzyme with upregulated expression was pyrophosphate fructose-6-phosphate 1-phosphotransferase, which performs the interconversion between fructose-6-phosphate and fructose-1,6-bisphosphate using pyrophosphate as an alternative phosphate donor for ATP. In a study on the transcriptome of high-density foci of *Giardia*-infected murine proximal intestine, the expression of this enzyme was not altered [8]. We found that the expression of a clone annotated GL50803_112103 (encoding arginine deiminase) was not induced during encystation and was not affected by the deficiency of the *glmyb2* gene (Fig. 5b). Arginine deiminase is one of three key enzymes of the arginine dihydrolase pathway and is highly activated in *G. lamblia* because of its ability to utilize arginine [39]. This result agrees with the published transcriptome data showing an unaltered or downregulated expression of these pathway components during encystation [6, 8]. Interestingly, a clone (GL50803_14651) encoding glucosamine 6-phosphate N-acetyltransferase was not the GlMyb2 target genes (Fig. 5b). It is out of prediction in that it is the second enzyme of N-acetyl galactosamine pathway highly expressing during encystation to supply UDP-N-acetyl galactosamine for *Giardia* cyst walls [8, 9]. The encystation method used in this study is the high bile method [29] which is efficient for cyst formation, but harsh to *Giardia* trophozoites. More convincing results could be obtained if we use other encystation protocols such as the method using cholesterol-deprivation [40], the two-step method using porcine bile and lactic acid [41], and the new method using a lower level of bile (5 mg/ml; [7]).

Another group of genes identified by ChIP-seq was categorized as signal transduction. Interestingly, three of five putative Nek clones were GlMyb2 target genes. Comparative kinome investigations with other excavates indicated that *Giardia* had the smallest known core kinases of any eukaryote, but it has a massively expanded Nek family, accounting for 198 of the 278 protein kinases in *Giardia* [42]. GlNek1 (GL50803_92498), showing encystation-induced expression, is involved in regulating the microtubule dynamics of the ventral disk [43]. In addition to GlNek1, the expression of eight putative GlNeks (GL50803_8350, GL50803_11364, GL50803_91451, GL50803_95593, GL50803_3957, GL50803_114307, GL50803_15409, and GL50803_133701) was found to be upregulated during encystation [5, 6, 42]. Our result indicated that GlNek (GL50803_133701) is another encystation-induced gene functioning in GlMyb2-mediated manner.

An additional GlMyb2 target gene encodes a homologous protein of SKD1, an AAA-type ATPase, that degrades plasma membrane proteins via the endosomal sorting complex required for transport pathway in plants [44]. Therefore, it is likely that the expression and function of *G. lamblia* SKD1 are required for encystation, in which the modulation of diverse secretory pathways occurs.

Finally, there was no information on the function of three genes identified as GlMyb2 target genes based on the homology database with amino acid sequences. The genome database of *G. lamblia* contains a set of abnormally high incidences of ORFs, which are annotated as hypothetical proteins because of the evolutional uniqueness of this parasite [45]. However, these hypothetical proteins do not necessarily have to be non-functional. Two of three genes encoding hypothetical protein (GL50803_21048, and GL50803_2926) showed encystation-induced expressions after all [5].

Although our ChIP experiments led to an isolation of GlMyb2-regulons, the efficiency was low because 20 out of 51 clones turned out to be genuine. More than 50% of clones did not have promoter region or show GlMyb2-dependent expressions during encystation (Fig. 5a; Table 1). The low efficiency may have been caused by omitting the pre-incubation with control pre-immune sera, isotype IgG, in ChIP-seq experiments. Apart from the well-known clones such as genes for cyst wall components and GlMyb2, it is difficult to predict the function of newly found clones in *Giardia* encystation because of lack of genetic and functional information.

The role of five putative binding sites for GlMyb2 were examined using the P_*glcwp1*_-luc reporter. Each of five GlMyb2 binding sites was mutated to examine whether the mutation affected the luciferase activity during encystation (Fig. 6). The promoters mutated at three GlMyb2 binding sites closer to the GlCWP1 ORF showed a decreased luciferase activity compared with that in wild-type promoter, even though all three constructs showed the encystation-induced expressions. In contrast, the mutations in the remaining two GlMyb2 binding sites did not affect luciferase activity in encysting cells. These results indicated that GlMyb2 interaction with a single binding site is not solely responsible for the encystation-induced expression of GlCWP1. Rather, the binding of multiple GlMyb2s may be required for its complete expression. For the efficient expression of *glcwp1*, the recruitment of other transcription machinery, including RNA polymerase, E2F1, WRKY, PAX2, and MBF1, is essential during encystation as suggested [25]. The effect of the cis-acting element mutation in the *glcwp1* promoter region could be pleiotropic by affecting the binding of these trans-acting components for *glcwp1* transcription.

## Conclusions

Through two sequential applications of CRISPR/Cas9 mutagenesis, four copies of *glmyb2* were completely removed from *Giardia* genome. The resulting *glmyb2* mutant was used to examine the authenticity of candidate GlMyb2 target genes obtained from ChIP-seq experiment. This provided the genetic evidence that GlMyb2 is one of the major transcription factors responsible for encystation in *Giardia*. Our results confirmed that GlMyb2 acts as a transcription activator, especially during encystation, by comparing the *glmyb2* mutant with wild type. Furthermore, the *glmyb2* null mutant could be used to elucidate the mechanism of how GlMyb2 plays a role in the life cycle of *G. lamblia*.

## Supplementary Information

**Additional file 1: Table S1.** Strains and plasmids used in this study.

**Additional file 2: Table S2** Primers used in this study.

**Additional file 3: Fig. S1.** Expression and localization of the SpCas9 protein in *Giardia*.** a** A schematic diagram of the expression plasmid for *Streptococcus pyogenes* Cas9 (SpCas9). The SpCas9 protein was expressed from P*gdh* (the promoter of *Giardia* glutamate dehydrogenase gene) with the 3′-untranslated region (3′-UTR) of *Giardia* α-tubulin gene in a fused form with nuclear localization signals (NLSs) of SV40 and *G. lamblia* protein (GL50803_2340). *Giardia *cells carrying this plasmid were selected by their puromycin resistance in which the puromycin N-acetyltransferase (*pac*) gene is expressed from P*ggir* (the promoter of *Giardia* γ-giardin gene) and the 3′-UTR of the *gdh* gene of *G. lamblia*. **b** Western blot analysis of *Giardia* carrying pKS-3HA.PAC (lane 1) or pSpCas9NLS.PAC (lane 2) using anti-SpCas9 antibodies (1:500). The same membrane treated in stripping buffer was then reacted with antibodies specific to the *G. lamblia* protein disulfide isomerase 1 (GlPDI1) (1:10,000). **c** An immunofluorescence assay of SpCas9-expressing *Giardia*. The *Giardia* cells fixed with chilled 100% methanol with PBS/0.5% Triton X-100 were reacted with mouse anti-SpCas9 antibodies (1:100) and then incubated with Alexafluor 488-conjugated anti-mouse IgG (1:100). The slides were mounted with ProLong™ Gold Antifade Mountant with DAPI for observation under an Axiovert 200 fluorescent microscope. Differential interference contrast (DIC) images showed cell morphology. Scale bars, 2 μm.

**Additional file 4: Fig. S2.** Screening of the *glmyb2* mutant strain JK1 by PCR analysis. *Giardia *trophozoites expressing SpCas9 were transfected with pgRNA-mybNEO, and the transfectants were selected by 600 μg/ml G418 via three sequential limiting dilutions on microtiter plates. Genomic DNA was prepared from the candidate mutant cells (8–20 wells per each gRNA) for the analysis of their *glmyb2* locus by PCR using primers Myb2-Det-F and Myb2-Det-R. **a** PCR products of 20 candidate mutants derived from CRISPR/Cas9 mutagenesis with gRNA, RG4. Genomic DNA prepared from wild-type *Giardia* WB is included as a control. The PCR products of wild-type and mutant *glmyb2* locus are indicated with arrows. **b** Percentages of cells showing three different patterns of PCR products (cells only showing wild-type PCR product, cells showing a mixed form of wild-type and mutant PCR DNAs, and cells only showing mutant PCR product) among the screened *Giardia* cells transfected with each gRNA for *glmyb2* mutagenesis and control gRNA.

**Additional file 5: Fig. S3.** Expression of five GlMyb2 target genes using luciferase reporters. **a** Construction of pNluc.PAC, the luciferase reporter plasmid without a promoter. The vector control plasmid lacks a promoter region in front of the NanoLuc luciferase (*nluc*) gene (Promega: pNL1.1), but it has 3′-UTR of Glα-tubulin gene and puromycin resistance (*pac*) cassette for expression in *G. lamblia*. **b** Expression of five GlMyb2-regulated genes using a luciferase reporter in wild-type (open bars) and *glmyb2* knockout mutant *G. lamblia* (closed bars). The promoter regions of five GlMyb2-regulated genes encoding GlCWP1 (GL50803_5638), GlCWP2 (GL50803_5435), GlCWP3 (GL50803_2421), HCNCP (GL50803_40376), and acetyl-CoA carboxylase/pyruvate carboxylase fusion protein (GL50803_113021) were cloned upstream of the *nluc* gene of pNluc.PAC. Cells carrying these plasmids were incubated in an encystation medium for 6, 15, and 24 h and used for luciferase assays. Luciferase activity was measured using a luminometer. Specific bioluminescence was calculated by normalizing the relative light units (RLU) with protein concentration. The luciferase activity of the wild-type and mutant cells carrying the empty vector, pNluc.PAC, was also monitored as a control. Three independent experiments were performed, and the data represent one of them showing a representative expression pattern.

## Data Availability

The data supporting the conclusions of this article are included within the article.

## Abbreviations

GlMyb2: *Giardia lamblia* Myb2
GlCWP: *Giardia lamblia* CWP
CRISPR: Clustered regularly interspaced short palindromic repeat
MLF: Myeloid leukemia factor
MBF1: Multi-protein bridging factor 1-like protein
ChIP: Chromatin immunoprecipitation
NLS: Nuclear localization signal sequence
gRNA: Guide RNA; PBS: phosphate-buffered saline
gDNA: Genomic DNA
GlPDI1: *Giardia lamblia* Protein disulfide isomerase 1
IFA: Immunofluorescence assay
NEO: Neomycin
ORF: Open reading frame
ESVs: Encysting specific vesicles
HCNCP: High cysteine non-variant cysteine protein
HCMPEGF: High cysteine-rich membrane proteins with epidermal growth factor repeats
Neks: Never-in-mitosis A-related kinases
