# Supplementary material for: Identification of target genes regulated by encystation-induced transcription factor Myb2 using knockout mutagenesis in Giardia lamblia
Source: Parasit Vectors. 2022 Oct 7;15:360. doi: 10.1186/s13071-022-05489-z (PMC9547401; doi:10.1186/s13071-022-05489-z)
Supplement: Supplementary file 1 — Additional file 1: Table S1. Strains and plasmids used in this study. [file 13071_2022_5489_MOESM1_ESM.docx]

**Table S1.** Strains and plasmids used in this study

| Organism /  Plasmid | Description^a^ | Source/  Reference |
| --- | --- | --- |
| *Giardia lamblia* |  |  |
| Wild type WB | Clinical isolate | ATCC  30957 |
| JK1 | Δ*myb2,* *myb2* deletion of 3 loci, neomycin resistant | This study |
| JK2 | Δ*myb2,* *myb2* deletion of 4 loci, neomycin and blasticidin resistant | This study |
| *Escherichia coli* |  |  |
| DH5α | *supE44, ΔlacU169 (Φ80 lacZ ΔM15), hsdR17, recA1, endA1, gyrA96, thi-1, relA1* | Invitrogen |
| BL21 (DE3) | *F9, ompT, hsdSB(rB-mB-) gal, dcm (DE3)* | Invitrogen |
| Rat |  |  |
| Sprague-Dawley | Crl:CD1 | Orient Bio |
| Plasmids |  |  |
| pP*gdh*-3HA.PAC | pKS-3HA.PAC, 150 bp promoter region of *Giardia* glutamate dehydrogenase gene (GiardiaDB accession # GL50803_21942) | [30] |
| pRGEN-Cas9-CMV | 4,167 bp encoding *Streptococcus pyogenes* Cas9 gene | ToolGen Inc. |
| pSpCas9.PAC | pP*gdh*-3HA.PAC, 4,167 bp encoding *Streptococcus pyogenes* Cas9 (SpCas9) gene | This study |
| pSpCas9NLS.PAC | pSpCas9.PAC, 105 bp 2340 nuclear localization signal (NLS) peptides (GiardiaDB accession # GL50803_2340) | This study |
| pUC57-gRNA | pUC57, guide RNA expression cassette | GenScript |
| pUC-Cont | pUC57-gRNA, control guide RNA expression cassette | This study |
| pUC-RG1 | pUC57-gRNA, RG1 guide RNA expression cassette | This study |
| pUC-RG2 | pUC57-gRNA, RG2 guide RNA expression cassette | This study |
| pUC-RG3 | pUC57-gRNA, RG3 guide RNA expression cassette | This study |
| pUC-RG4 | pUC57-gRNA, RG4 guide RNA expression cassette | This study |
| pKS-3HA.NEO | Shuttle vector, Amp^R^, *neomycin* resistance (*neo*) cassette | [31] |
| pBluescript II SK | Cloning vector, Amp^R^ | Clontech |
| pSK-mybNEO | pBluescript II SK, *neo* cassette between 1 Kb upstream and downstream of *G. lamblia* *myb2* (*glmyb2*, GiardiaDB accession # GL50803_8722) gene | This study |
| pCont-mybNEO | pSK-mybNEO, control guide RNA expression cassette | This study |
| pRG1-mybNEO | pSK-mybNEO, control guide RNA expression cassette | This study |
| pRG2-mybNEO | pSK-mybNEO, control guide RNA expression cassette | This study |
| pRG3-mybNEO | pSK-mybNEO, control guide RNA expression cassette | This study |
| pRG4-mybNEO | pSK-mybNEO, control guide RNA expression cassette | This study |
| pRG1-dmybNEO | pUC-RG1, *neo* cassette between 1 Kb upstream and downstream of the narrow *glmyb2* gene | This study |
| pKS-3HA.BSR | Shuttle vector, Amp^R^, blasticidin-S resistance (*bsr*) cassette | [31] |
| pRG1-dmybBSR | pUC-RG1, *bsr* cassette between 1 Kb upstream and downstream of the narrow *glmyb2* gene | This study |
| pKS-3HA.PAC | Shuttle vector, Amp^R^, puromycin N-acetyltransferase resistance (*pac*) cassette | [31] |
| pPmyb2HA.PAC | pKS-3HA.PAC, 150 bp own promoter region with 1,590 bp encoding *glmyb2* gene | This study |
| pET32a | Expression vector, Amp^R^ | Novagen |
| pET-myb2 | pET32a, 1,590 bp encoding *glmyb2* | This study |
| pNL1.1 | NanoLuc luciferase (*nluc*) gene containing vector | Promega |
| pNluc.PAC | pKS-3HA.PAC, *nluc* encoding gene without a promoter | This study |
| pP5638-Nluc.PAC | pNluc.PAC, 150 bp promoter region of cyst wall protein 1 (*cwp1*, GiardiaDB ID GL50803_5638) | This study |
| pP5435-Nluc.PAC | pNluc.PAC, 151 bp promter region of cyst wall protein 2 (GiardiaDB ID GL50803_5435) | This study |
| pP2421-Nluc.PAC | pNluc.PAC, 150 bp promter region of cyst wall protein 3 (GiardiaDB ID GL50803_2421) | This study |
| pP40376-Nluc.PAC | pNluc.PAC, 150 bp promter region of high cysteine non-variant cyst protein (GiardiaDB ID GL50803_40376) | This study |
| pP113021-Nluc.PAC | pNluc.PAC, 151 bp promter region of acetyl-CoA carboxylase/pyruvate carboxylase fusion protein (GiardiaDB ID GL50803_113021) | This study |
| pP5638-100 | pNluc.PAC, 100 bp promoter region of *cwp1* gene | This study |
| pP5638-75 | pNluc.PAC, 75 bp promoter region of *cwp1*gene | This study |
| pP5638-50 | pNluc.PAC, 50 bp promoter region of *cwp1* gene | This study |
| pP5638-25 | pNluc.PAC, 25 bp promoter region of *cwp1*gene | This study |
| pP5638-mt1 | pNluc.PAC, mutated 75 bp promoter region of *cwp1* gene | This study |
| pP5638-mt2 | pNluc.PAC, mutated 75 bp promoter region of *cwp1* gene | This study |
| pP5638-mt3 | pNluc.PAC, mutated 75 bp promoter region of *cwp1* gene | This study |
| pP5638-mt4 | pNluc.PAC, mutated 100 bp promoter region of *cwp1* gene | This study |
| pP5638-mt5 | pNluc.PAC, mutated 100 bp promoter region of *cwp1* gene | This study |

^a^ Amp, ampicillin; ^R^, resistant
