# Supplementary material for: Identification of target genes regulated by encystation-induced transcription factor Myb2 using knockout mutagenesis in Giardia lamblia
Source: Parasit Vectors. 2022 Oct 7;15:360. doi: 10.1186/s13071-022-05489-z (PMC9547401; doi:10.1186/s13071-022-05489-z)
Supplement: Supplementary file 2 — Additional file 2: Table S2 Primers used in this study. [file 13071_2022_5489_MOESM2_ESM.docx]

**Table S2.** Primers used in this study

| Name | Nucleotide sequence (5'-3')^a^ |
| --- | --- |
| **For SpCas9 expressing constructs** | |
| SpCa9-F | GTTACGCGGCCGCATGGACAAGAAGTACAGCATCGGC |
| SpCas9-R | AGCTCTAGAGGCGTAGTCGGGCACGTCGTAGG |
| 2340-F | AGCTCTAGACCAGAGAAGAAGCGGTCCAAGTC |
| 2340-R | GGATCGATGCTCTTAATTTTACTAACTCTA |
| **For *myb2* gene deletion constructs** | |
| neo-cassette-F | GCGGGATCCGTGATCAAATGCCTTCGAGCGT |
| neo-cassette-R | CATAGGTACCCCAGCTGATCGGCGCCGGGAT |
| myb2-up-F | GCTAGGGCCCACGCAATCGGAGTCATTGGCTT |
| myb2-up-R | GCGGGATCCAAAATCATAGACTTTCAAGAAAT |
| myb2-down-F | CATAGGTACCCGACGTCACTGACTTTCAAAGT |
| myb2-down-R | CTTACGCGGCCGCGCTTCAATTCTGCGCATCCATC |
| myb2NEO-F | CGCGAATGCATCTAGACGCAATCGGAGTCATTGG |
| myb2NEO-R | TGATTACGCCAAGCTGCTTCAATTCTGCGCATCCATCATGTC |
| 2nd-myb2-up-F | TCTAGATATCGGATCCTTGCCTACTGATGGTTTGTCTAACACAAC |
| 2nd-myb2-up-R | TTGATCACTGTTGATGGCTGAGAAGGTACCG |
| 2nd-myb2-down-F | CAGCTGGGCAACATCCTTCCCCGTGAG |
| 2nd-myb2-down-R | GGCCTCTGCAGTCGACCTACAAGTGACGACACCAAACTTGC |
| dmyb-F | GGGCTGAAGTGAATATTTACCTTTTCCG |
| dmyb-R | GATTTTCTTGTTTTAGGGTTAGTTTTTTAGAAAAGGC |
| bsr-F | CCCTAAAACAAGAAAATCatgggaatgaaaacatt |
| bsr-R | AAATATTCACTTCAGCCCtcactcgactca |
| myb2-Det-F | AGAAGGATCAAACTTATCTAGATA |
| myb2-Det-R | ACATCTGGCGAAGGAGAAGTTGA |
| Det2-F | CCGAAACGCGTACCCCAGCCAA |
| Det2-R | GCTTCAGTAGGGTTTTTGCCAGTCT |
| **For complementary construct** | |
| Pmyb2-F | GTTACGCGGCCGCTTCTCTTCAGTCAACGCCATTA |
| myb2-R | CATA CTCGAG GGGTAGCTTCTCACGGGGAAGGAT |
| **For real-time PCR** |  |
| myb2-RT-F | TTAATTGAGGCGGTCAAGCTT |
| myb2-RT-R | GGGTAGCTTCTCACGGGGAA |
| actin-RT-F | GTCCGTCATACCATCTGTTC |
| actin-RT-R | GTTTCCTCCATACCACACG |
| 5638-RT-F | GGCTTTCGGGAGACTCTATTTG |
| 5638-RT-R | ATGTTCTCCGGGATGGTACCT |
| 5435-RT-F | GGCCTCAACGTCCCGCTCC |
| 5435-RT-R | CACCTTCTGCGGACAATAG |
| 113531-RT-F | GCCAGTGCATCTCGGAT |
| 113531-RT-R | CCGAAAGAATGCTCTCGT |
| 114626-RT-F | GGCTTCGCTGAATAACCTCT |
| 114626-RT-R | GAGCTGGTCAAGAACACTT |
| 40376-RT-F | CGTTCAGGGTTGTCGCATT |
| 40376-RT-R | TTACACAGCTTTGCTCCTAC |
| 137701-RT-F | AGGAATAATGGCATACGTTGC |
| 137701-RT-R | CAGGTCCGGCCTCCACCCGTCA |
| 21924-RT-F | ATAACTGCAATACGTAATAA |
| 21924-RT-R | CTAAGAGTCATTGTGGCCGC |
| 114495-RT-F | GCGTGCTGCGCATACGAACG |
| 114495-RT-R | TACATCAGAGTAGTTGCTG |
| 42657-RT-F | TTGGCACGCTTGAAGGCCG |
| 42657-RT-R | AATCTTTCTCGTTGGCCAG |
| 13109-RT-F | CCGGGCGTTCGCTGCGCT |
| 13109-RT-R | CTAACAGTAGCACCGTGAAG |
| 7512-RT-F | TTACTGTATCCTGTGGGTGT |
| 7512-RT-R | TCACTGCTTTGCGGTGGAAG |
| 6664-RT-F | CATAATGTTTGTTGCGGGG |
| 6664-RT-R | TCACTTCTCCCCCACGACCC |
| 15469-RT-F | GCCGGAAAACAAGGCCCTTCGT |
| 15469-RT-R | CTATGAGTGCTTAAACTGC |
| 112103-RT-F | ATCACGTGGAGGATGCCGTC |
| 112103-RT-R | TCACTTGATATCGACGCAGA |
| 14993-RT-F | CAGATGACCGACCTGAATGG |
| 14993-RT-R | TTACTTTCGAGCCTCTTCGA |
| 14651-RT-F | GTCGTAGATCTTACTCTTCG |
| 14651-RT-R | AGAAATAAATAGCCATCA |
| 113021-RT-F | ACCAGGGGTTGTGGAGAAGC |
| 113021-RT-R | TCAGCTTACCACGAGAAGAA |
| 7875-RT-F | TTCAATCTGTTCTACACGAG |
| 7875-RT-R | TTACAAGATGCAGGACTGGT |
| 16078-RT-F | TTTCTCCGAACGCTTCCTC |
| 16078-RT-R | CTAGCGTCCGTTATTGTAAT |
| 2926-RT-F | CGCACCAAGCTACGGGAGT |
| 2926-RT-R | CTATTCTAGAAGGTCTGAT |
| **For recombinant protein** | |
| rmyb2-F | CGGGAGCTCATGTTACCGGTACCTTCTCAG |
| rmyb2-R | TTAGCGGCCGCAGGGTAGCTTCTCACG |
| **For luciferase assay** | |
| KS-F | tctttgcatcatcgttgctcggt |
| KS-R | cttaagctcgaggtcgacggtatc |
| Nluc-F | gacctcgagcttaagCATGGTCTTCACACTCGAAGATTTCG |
| Nluc-R | acgatgatgcaaagaGCCTTACGCCAGAATGCG |
| 2421-F | GTACCGAGCTCATGCATACCACTCCGGCGGC |
| 2421-R | TGCCTAAGCTTATCAGTAGTAACTTATTTTTTG |
| 2926-F | GTACCGAGCTCGTACCGTCTAGCGTTAAGTCT |
| 2926-R | TGCCTAAGCTTCTTCGCTAATCTACAGTTTAC |
| 5435-F | GTACCGAGCTCTACAACCCACTTTGATGAGAG |
| 5435-R | TGCCTAAGCTTTTTATTTTCCCAGCCACTGTT |
| 5638-F | GTACCGAGCTCGACAACGGCTTACTAAATCA |
| 5638-R | TGCCTAAGCTTCCCTGATATTTTATTTCTG |
| 40376-F | GTACCGAGCTCAACTCTCACCGTGTCACAGG |
| 40376-R | TGCCTAAGCTTTTTTGTTTTGTGGGCATCTACTG |
| 113021-F | GTACCGAGCTCTTAGCACTTACTTGTTCGGT |
| 113021-R | TGCCTAAGCTTGTTCGATATCAGAAAAT |
| 5638-100-F | GTACCGAGCTCTGCATTTTTTCTGGCTAACAGT |
| 5638-75-F | GTACCGAGCTCCAGTCTACAATTTACAGTATA |
| 5638-50-F | GTACCGAGCTCCTACAGTTTACAACTCTCAGAT |
| 5638-25-F | tacgactcactatagggcgaattggagctCGAAACACAGAAATAAAATATCAGGGAagcttatcga |
| 5638-25-R | gatgcaaagGTCGACGGAAAgaattcTTACGCCAGAATGCGTTCGC |
| 5638-mt1-F | GTACCGAGCTCCAGTCTACAATTTACAGT***TATGT****T*T |
| 5638-mt2-F | GTACCGAGCTCCAGTCTACAAT***AATGTC***TATACAAT |
| 5638-mt3-F | GTACCGAGCTCCAGT***GATGTT***TTTACAGTATACAAT |
| 5638-mt4-F | GTACCGAGCTCTGCATTTTTTCTGGCTAACAGT***GATGTC***T |
| 5638-mt5-F | GTACCGAGCTCTGCATTTTTTCTGGC***ATTGTC***T |

^a^ Restriction enzyme sites are underlined and ^b^ Mutation sequences are shown in bold italic.
