# Supplementary figures and images for: Identification of target genes regulated by encystation-induced transcription factor Myb2 using knockout mutagenesis in Giardia lamblia
Source: Parasit Vectors. 2022 Oct 7;15:360. doi: 10.1186/s13071-022-05489-z (PMC9547401; doi:10.1186/s13071-022-05489-z)

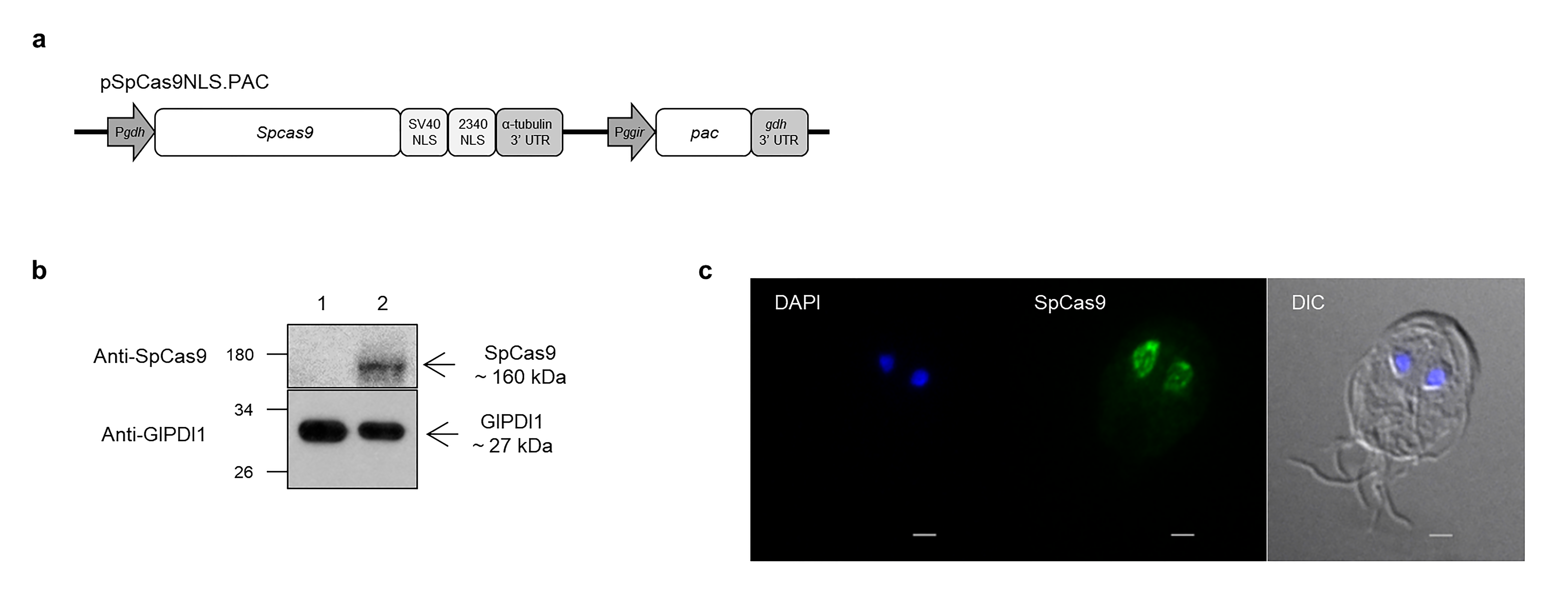

Supplement: Supplementary file 3 — Additional file 3: Fig. S1. Expression and localization of the SpCas9 protein in Giardia. a A schematic diagram of the expression plasmid for Streptococcus pyogenes Cas9 (SpCas9). The SpCas9 protein was expressed from Pgdh (the promoter of Giardia glutamate dehydrogenase gene) with the 3′-untranslated region (3′-UTR) of Giardia α-tubulin gene in a fused form with nuclear localization signals (NLSs) of SV40 and G. lamblia protein (GL50803_2340). Giardia cells carrying this plasmid were selected by their puromycin resistance in which the puromycin N-acetyltransferase (pac) gene is expressed from Pggir (the promoter of Giardia γ-giardin gene) and the 3′-UTR of the gdh gene of G. lamblia. b Western blot analysis of Giardia carrying pKS-3HA.PAC (lane 1) or pSpCas9NLS.PAC (lane 2) using anti-SpCas9 antibodies (1:500). The same membrane treated in stripping buffer was then reacted with antibodies specific to the G. lamblia protein disulfide isomerase 1 (GlPDI1) (1:10,000). c An immunofluorescence assay of SpCas9-expressing Giardia. The Giardia cells fixed with chilled 100% methanol with PBS/0.5% Triton X-100 were reacted with mouse anti-SpCas9 antibodies (1:100) and then incubated with Alexafluor 488-conjugated anti-mouse IgG (1:100). The slides were mounted with ProLong™ Gold Antifade Mountant with DAPI for observation under an Axiovert 200 fluorescent microscope. Differential interference contrast (DIC) images showed cell morphology. Scale bars, 2 μm. [file 13071_2022_5489_MOESM3_ESM.tif]

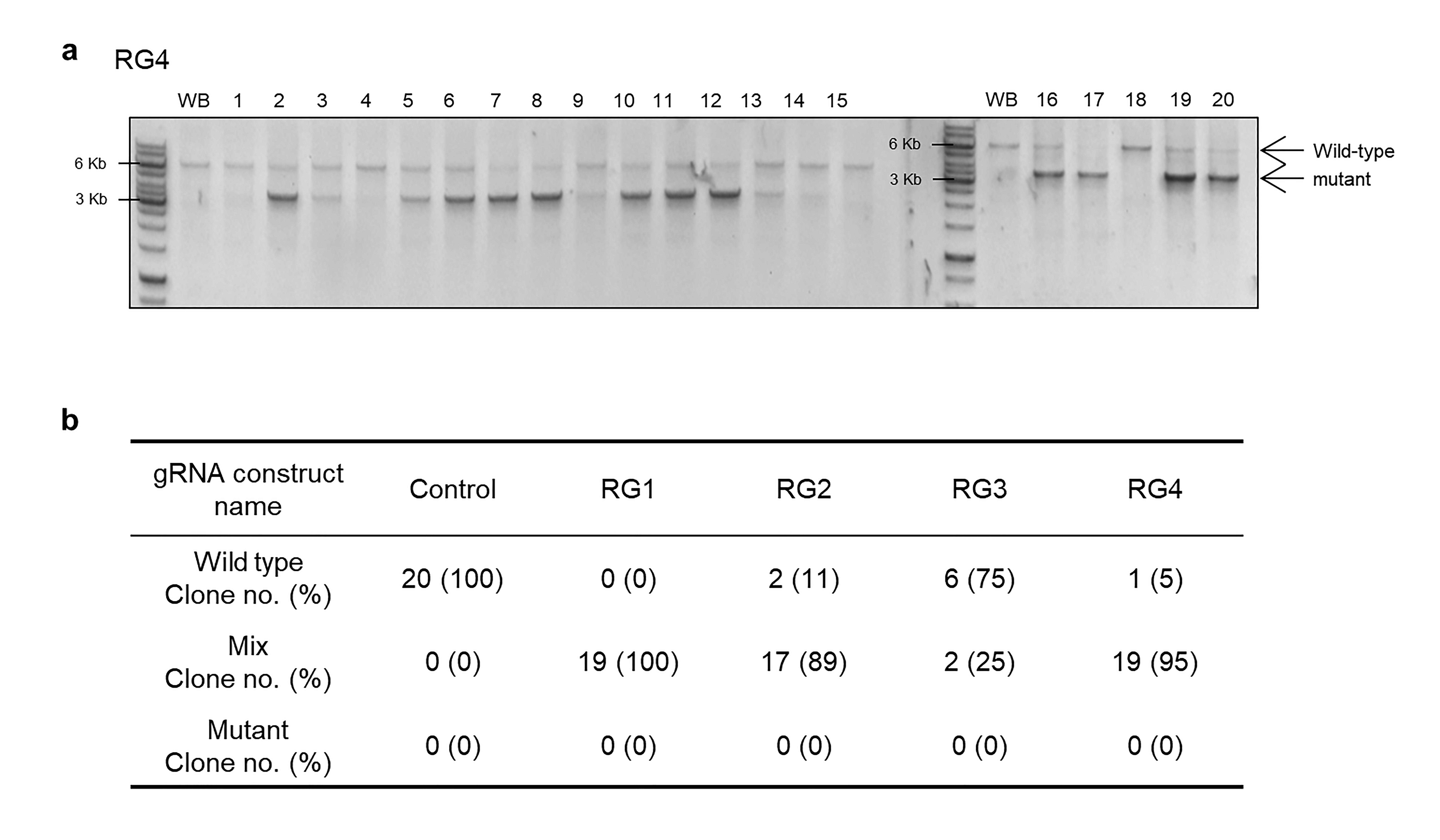

Supplement: Supplementary file 4 — Additional file 4: Fig. S2. Screening of the glmyb2 mutant strain JK1 by PCR analysis. Giardia trophozoites expressing SpCas9 were transfected with pgRNA-mybNEO, and the transfectants were selected by 600 μg/ml G418 via three sequential limiting dilutions on microtiter plates. Genomic DNA was prepared from the candidate mutant cells (8–20 wells per each gRNA) for the analysis of their glmyb2 locus by PCR using primers Myb2-Det-F and Myb2-Det-R. a PCR products of 20 candidate mutants derived from CRISPR/Cas9 mutagenesis with gRNA, RG4. Genomic DNA prepared from wild-type Giardia WB is included as a control. The PCR products of wild-type and mutant glmyb2 locus are indicated with arrows. b Percentages of cells showing three different patterns of PCR products (cells only showing wild-type PCR product, cells showing a mixed form of wild-type and mutant PCR DNAs, and cells only showing mutant PCR product) among the screened Giardia cells transfected with each gRNA for glmyb2 mutagenesis and control gRNA. [file 13071_2022_5489_MOESM4_ESM.tif]
